# Normal Pressure Hydrocephalus in Adult Mice Causes Gait Impairment, Cognitive Deficits, and Urinary Frequency with Incontinence

**DOI:** 10.1523/ENEURO.0412-24.2024

**Published:** 2024-11-26

**Authors:** Margaret M. Tish, Natalie A. Voss, Aimee X. Bertolli, Miles J. Klimara, Richard J. Smith, Daniel R. Thedens, Chantal Allamargot, Marco M. Hefti, Matthew A. Howard, Georgina M. Aldridge, Joel C. Geerling

**Affiliations:** ^1^Department of Neurology, University of Iowa, Iowa City, Iowa 52246; ^2^Iowa Neuroscience Institute, Iowa City, Iowa 52246; ^3^Departments of Otolaryngology, University of Iowa, Iowa City, Iowa 52246; ^4^Radiology, University of Iowa, Iowa City, Iowa 52246; ^5^Central Microscopy Research Facility, University of Iowa, Iowa City, Iowa 52246; ^6^Departments of Pathology, University of Iowa, Iowa City, Iowa 52246; ^7^Neurosurgery, University of Iowa, Iowa City, Iowa 52246

**Keywords:** Hakim’s triad, gait apraxia, kaolin, magnetic gait, micturition, urinary urgency incontinence

## Abstract

Normal pressure hydrocephalus (NPH) is marked by enlarged cerebral ventricles with normal intracranial pressure, plus three stereotypical symptoms: gait impairment, cognitive dysfunction, and urinary frequency with urge incontinence. The neural circuit dysfunction responsible for each of these symptoms remains unknown, and an adult mouse model would expand opportunities to explore these mechanisms in preclinical experiments. Here, we describe the first mouse model of chronic, communicating hydrocephalus with normal intracranial pressure. Hydrocephalic male and female mice had unsteady gait and reduced maximum velocity. Despite performing well on a variety of behavioral tests, they exhibited subtle learning impairments. Hydrocephalic mice also developed urinary frequency, and many became incontinent. This mouse model, with symptoms resembling human NPH, can be combined with molecular-genetic tools in any mouse strain to explore the neural circuit mechanisms of these symptoms. Preclinical work using this hydrocephalus model will lead to the development of new treatments for NPH symptoms.

## Graphical abstract

**Figure:**
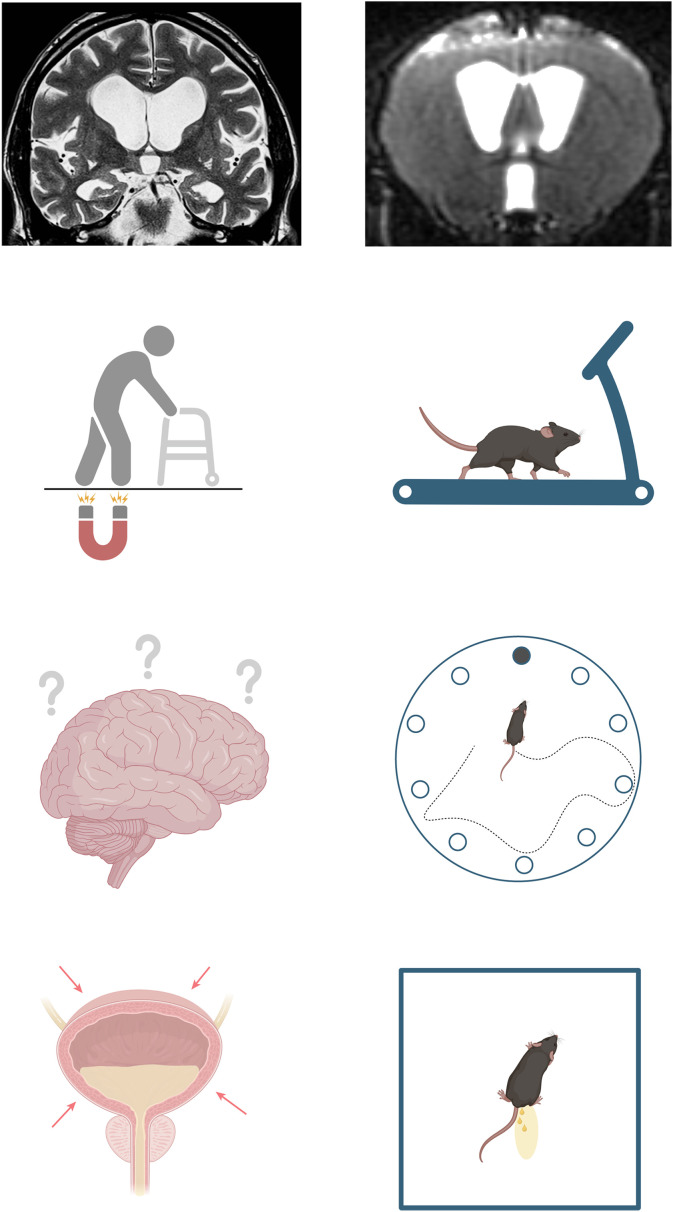
Visual Abstract

## Significance Statement

Like human patients with normal pressure hydrocephalus (NPH), mice with communicating hydrocephalus develop enlarged cerebral ventricles with normal intracranial pressure plus three stereotypical symptoms: gait impairment, cognitive dysfunction, and urinary frequency with incontinence. This mouse model, with symptoms resembling human NPH, can be combined with molecular-genetic tools in any mouse strain to explore neural circuit mechanisms of NPH symptoms.

## Introduction

Normal pressure hydrocephalus (NPH) was first described by Hakim and Adams in 1965. They identified a series of adult patients with enlarged cerebral ventricles despite normal cerebrospinal fluid (CSF) pressure. These patients had three characteristic symptoms: gait impairment, cognitive dysfunction, and urinary incontinence (Hakim and Adams, 1965). Similar to the original description, current diagnostic criteria for NPH require enlarged ventricles, normal CSF pressure, and at least one of the three characteristic symptoms (Marmarou et al., 2005; Mori et al., 2012; Damasceno, 2015). By age 70, 1.5% of adults meet these diagnostic criteria and 5.4% have neuroimaging findings that suggest NPH (Constantinescu et al., 2024).

Some NPH symptoms improve after inserting a shunt into the cerebral ventricles to drain excess CSF (Adams et al., 1965; Hakim and Adams, 1965; Shenkin et al., 1973; Williams and Malm, 2016). However, not all symptoms respond equally, with gait responding most often (Hebb and Cusimano, 2001; Klinge et al., 2005; Pujari et al., 2008; Toma et al., 2013). The pathophysiology and neural circuit mechanisms of NPH symptoms remain unknown, and there are no effective treatments for shunt-resistant symptoms—many patients require a wheelchair or adult diapers. The scarcity of experimental animal models limits opportunities to investigate neurologic mechanisms and develop new treatments, but opportunities could increase if a mouse model of NPH becomes available.

Combining an NPH disease model with molecular-genetic tools already available in mice would accelerate progress in unraveling the neurologic mechanisms of NPH symptoms. In 1913, Dandy and Blackfan created the first animal model of hydrocephalus by blocking the cerebral aqueduct in dogs (Dandy and Blackfan, 1913, 1914). Various forms of hydrocephalus have been modeled in other species (Hochwald, 1985; Di Curzio, 2018; Chen et al., 2023), but we are not aware of an adult mouse model for NPH. NPH is a chronic illness of older adults, yet most rodent studies use neonatal or juvenile animals to model pediatric hydrocephalus (Del Bigio et al., 1997; Khan et al., 2006; Lopes Lda et al., 2009). Also, NPH is a “communicating” hydrocephalus, meaning that CSF flow between ventricles is preserved (Hakim and Adams, 1965; Fisher, 1977; Oliveira et al., 2019). While interventricular communication remains patent in some models (Borit and Sidman, 1972; Bloch et al., 2006; Li et al., 2008; Jusué-Torres et al., 2016), other methods produce “noncommunicating” hydrocephalus, with obstructed interventricular flow (Dandy and Blackfan, 1913; Matsumoto et al., 1975; Khan and Del Bigio, 2006).

Importantly, while some studies have tested gait and cognitive function, none have assessed the third characteristic symptom, micturition (Kuchiwaki et al., 1994; Del Bigio et al., 1997, 2012; F. E. Olopade et al., 2012; Jusué-Torres et al., 2016). Human NPH patients often develop urinary frequency and become incontinent, but no information is available regarding urinary continence in hydrocephalic animal models. An ideal NPH model would include chronically enlarged cerebral ventricles with normal CSF pressure in adult animals, plus one or more of the characteristic NPH symptoms.

Here, we used a multimodal approach to test the effects of chronic, communicating hydrocephalus on intracranial pressure, gait, memory, and micturition in adult mice. We found that hydrocephalic mice survive for months, have normal intracranial pressure, and perform normally on many behavioral tests. Like human patients, they develop gait impairment, cognitive dysfunction, and urinary frequency with incontinence. This mouse model can be combined with a variety of mouse genetic models and neuroscience tools to explore the neural circuit mechanisms of NPH symptoms.

## Materials and Methods

### Mice

All mice were group housed in a temperature- and humidity-controlled room on a 12 h light/dark cycle with *ad libitum* access to water and standard rodent chow, except as described for specific experimental assays. We used C57BL/6J mice (The Jackson Laboratory) and mixed-background littermates from breeding colonies in our laboratory, all of which were bred on a C57BL6/J genetic background. Overall, we used *n* = 213 adult male and female mice, ranging in age from 2 to 8 months (on average 116 ± 44 d) and 20–40 g body weight at the beginning of each experiment. Behavioral tests described below were performed on separate days with typically at least 1 d of rest in the home cage between tests.

### Kaolin preparation

Kaolin (synthetic aluminum silicate, 82% SiO_2_; Sigma-Aldrich #09772) was prepared fresh on the day of each surgery. Three different suspensions were prepared by adding the appropriate amount (0.10 g for 10%, 0.125 g for 12.5%, and 0.15 g for 15%) to 1 ml of sterile saline. Immediately prior to each injection, the tube containing this mixture was shaken vigorously to resuspend kaolin.

### Surgery

Mice were anesthetized with isoflurane (0.5–2.0%), injected with an analgesic drug (carprofen or meloxicam, 1 mg/kg, s.c.) and placed in a stereotactic frame (Kopf 1900 or 940). The head was rotated to a nearly 90° downward angle, and the snout was placed in a custom-built, vertically oriented nose cone for continuous anesthetic gas delivery. We made a midline incision and retracted the skin to expose the midline at the base of the occipital bone. The muscles were then separated to expose the atlanto-occipital membrane. A Hamilton syringe with a custom needle (20° bevel, 25 mm; Hamilton #7803-07) was positioned directly over the center of the membrane, with the bevel facing caudally (away from the cerebellum). We injected 5 µl of sterile 0.9% saline, kaolin (10, 12.5, or 15% suspension), or LPS (1, 3, or 5 µg/kg in sterile saline), or 1–10 µl of analogous blood (drawn from the base of the tail immediately before injection). In each case, the solution or suspension was first loaded into the Hamilton syringe using an insulin syringe (28 ga; BD #329410). The Hamilton syringe and needle were then lowered until the entire bevel was under the atlanto-occipital membrane before gradual injection into the cisterna magna in 0.5–1 µl steps over ∼30 s. The needle was left in place for 5 min and then removed. The scalp incision was then closed with Vetbond (3M) and the mouse was moved to a warm recovery cage. All mice received a second analgesic injection of carprofen the day after surgery. For at least 5 d and typically 2 weeks or longer following surgery, mice were checked daily. Any mice appearing dehydrated were given 0.3–0.75 ml of sterile 0.9% saline subcutaneously, and a Napa Nectar gel pack was place on the cage floor. If a mouse lost >30% of its body weight or had a body condition score (BCS) health score of 1–2 (out of 5), we killed the mouse and counted it as an early/unplanned death in the overall Kaplan–Meier analysis.

In a series of pilot tests, we injected a range of different kaolin concentrations (10–20%) and found that a 10% suspension (*n* = 9) did not consistently produce hydrocephalus, while 20% did not remain in suspension well enough for pilot injections (Extended Data Fig. 2-1). Based on this information, we injected 5 µl of either 12.5% (*n* = 7) or more often 15% (*n* = 113) kaolin into the cisterna magna for most experiments in this study. Kaolin-injected mice that did not develop hydrocephalus, defined as an overall cerebral ventricular volume more than three standard deviations above the mean of unoperated mice, were excluded from further analysis. All hydrocephalic mice (regardless of concentration) were analyzed together because, after excluding nonhydrocephalic mice, the ventricular volumes of groups injected with 10, 12.5, or 15% kaolin were not significantly different by ANOVA.

In five additional mice, we tested patency of interventricular foramina by injecting Evans blue dye (1% in sterile water) into the lateral ventricle 4 weeks after kaolin injection. After anesthetizing each mouse and placing it into a stereotaxic apparatus as above (but using a standard, horizontal isoflurane nose cone), and after exposing the skull as above, we used a 0.8 mm drill bit to produce a burr hole at a stereotaxic coordinate 0.8 mm lateral and 0.25 mm rostral to the bregma. Next, a needle was inserted slowly into the right lateral ventricle to reach 2.6 mm deep to bregma. We injected 3 µl of dye gradually, over 30 s. After a delay of up to 15 min (to allow dye to equilibrate and flow through the cerebral ventricular system), the mouse was anesthetized with ketamine–xylazine and perfused as described below (see Perfusions and histology). After perfusion, each brain was removed and inspected for the presence of blue dye in the basilar cisterns and periarterial subarachnoid spaces.

### Intracranial pressure recordings

Initially, to test ICP, we connected a short-bevel 20 gauge needle via silastic tubing to a board-mount pressure sensor (Honeywell HSCSAAN010NDAA5), similar to a previously published approach (Moazen et al., 2016). We connected the pressure transducer to a DI-1100 analog-to-digital converter (DATAQ) and monitored output voltage using WinDaq software. Before each mouse recording, we calibrated the system using a graduated cylinder to measure voltage output at 2.5, 5, 7.5, 10, and 15 cmH_2_O (similar to Moazen et al., 2016). After anesthetizing each mouse and placing it in a stereotaxic frame as above, a 0.8 mm drill bit was used to create a burr hole in the skull over the right lateral ventricle, without impacting the dura. The needle was then lowered slowly through the dura and into the ventricle, using the coordinates above. Voltage output from the sensor was recorded and later compared with calibration data obtained as above to convert voltage readings to pressure in cmH_2_O. The initial ICP measurement approach was used in *n* = 4 experimental mice per group at 1, 2, 3, and 9 weeks after kaolin injection, as well as *n* = 3 control mice 9 weeks after saline injection.

Subsequently, to obtain higher-resolution ICP recordings, we used a different pressure sensor (Millar SPR-671). Before each recording, the sensor was soaked in 37°C sterile water for at least 30 min and then calibrated with the tip in 37°C water, at 0 and 10 mmHg, using a mercury manometer. Mice were anesthetized, prepped, and placed in a stereotaxic frame, as above. After incising the scalp to expose and level the skull, a 0.8 mm bit was used to drill a burr hole above the right lateral ventricle (0.8 mm lateral and 0.25 mm rostral to bregma). The pressure sensor was then lowered slowly (30 s) through the burr hole to a depth of 2.6 mm below bregma. The pressure sensor was connected to an ADInstruments bridge amplifier via an AEC-10D cable, and the digitized intracranial pressure signal was monitored and recorded in LabChart (ADInstruments). After confirming cardiac ± respiratory modulation and recording a stable ICP for 3–5 min, followed by application of gentle abdominal pressure and several additional minutes of recording, the pressure sensor was removed slowly (over ∼1 min), cleaned with Haemo-Sol enzymatic cleaner, and placed in 37°C water for ∼15 min before recalibration. We calculated the 10 s average ICP from every recording after the probe had been inserted for 30 s. To calculate amplitude, three waves within 20–50 s after inserting the sensor were chosen at random and their trough and peak pressure values extracted.

### Magnetic resonance imaging

Mice were anesthetized with isoflurane and placed in a 7 T small animal MR imager (MR901, GE) equipped with a volume transmit coil and two-channel mouse head surface coil. The brains were scanned using a standard T2-weighted fast spin echo pulse sequence with echo time 65–85 ms, in-plane resolution 0.1 mm, and slice thickness 0.5 mm with independent acquisitions in the horizontal, sagittal, and coronal planes. After acquisition, all DICOM images were analyzed in 3D Slicer. A threshold was set to highlight ventricular CSF and minimize parenchymal tissue signal. Any remaining nonventricular labeling in the brain parenchyma (typically in the orbit and skull) was then erased manually until only the cerebral ventricles were highlighted. A 3D model was then saved, and the total ventricular volume calculated and recorded. Separate volumes were calculated for the lateral, third (plus aqueduct), and fourth ventricles of each mouse by manually erasing the other ventricles on saved copies of the original volume.

### Rotarod

For habituation, each mouse was placed atop a stationary rod (Rotamex, Columbus Instruments) for 30 s. If a mouse fell during this time, they were put back on the rod. Next, the rod began rotating at 4 rpm for 60 s. This habituation test was repeated three times per day for 2 d. We began testing mice the following day and retested each of the 3 weeks before saline or kaolin injection and each of the 8 weeks after surgery. Each week, mice were tested on 2 consecutive days, with three tests per day. Results were averaged for each week. Once each mouse was placed atop the unroating rod, a preprogrammed protocol was started. Male mice were run using a protocol in which the rod began rotating at 4 rpm and increased by 1.2 rpm every 20 s. Female mice were run using a slightly slower protocol that increased by 1.0 rpm every 20 s. The program stopped after 600 s (40 rpm). The time and speed at which the mouse fell were recorded.

### DigiGait

Mice were tested in a DigiGait treadmill apparatus (Mouse Specifics) 4 weeks after saline or kaolin injection. In initial experiments, we attempted to test mice repeatedly, both before and after surgery, but learned that most mice would no longer attempt to walk in this apparatus after the first test session. Therefore, we repeated the experiment, only testing mice once. Videos were recorded of mice walking at a treadmill speed of 10 cm/s for at least 5 s without stopping or turning the head sharply. Videos were then trimmed in the DigiGait imager, selecting the best 3–5 s of continuous, consistent movement. Videos were then imported into DigiGait analysis software, manually edited to remove improperly identified paws and to connect incorrect breaks in each step. The analyzed data were exported to Microsoft Excel, where we compared stride time, stride length, strides per second, and swing time between control and hydrocephalic mice. After capturing initial videos for DigiGait analysis, we used the same treadmill to assess maximum gait speed by increasing belt speed by 1 cm/s until the mouse could no longer keep up (still running but not fast enough to avoid slipping into back bumper).

### Micturition video thermography

Using a noninvasive, thermal imaging approach we have described in detail previously (Verstegen et al., 2020), we tested micturition in male mice every week for 2–3 weeks before and through 10 weeks after injection of kaolin or saline. In subsequent tests using hydrocephalic female mice, due to the lack of an apparent change in the initial few weeks in male mice, we ran weekly MVT tests for 2 weeks before and again for Weeks 5 through 10 after injection.

A microbolometer-style thermal camera (FLIR A35) was placed 2 feet above a table surface with up to four acrylic enclosures. Schematics for these enclosures, with down-sloping walls designed to give the thermal camera a full view of all cage corners, are available in a previous publication (Verstegen et al., 2020). Between the enclosure and table was a large sheet of blotting paper (Blick #10422-1005). One mouse was placed in each cage for a 2 h recording. To avoid “marking” behavior that occurs rarely, in a subset of dominant male mice, male mice were always run alongside others from the same home cage.

After recording, every thermal recording was analyzed using ResearchIR (FLIR). Voided urine is initially well above room temperate (bright on thermal imaging) and then cools below room temperature within 1–2 min. Typical voids stay below room temperature (dark on thermal imaging) for more than an hour, and the surface area of the “void spot” is proportional to the volume of voided urine. Between 10 s after all mice have been placed in their enclosures and 2 h later, when they are returned to their home cages, we record the timing and location of every void and describe mouse behavior around the time of the void. The first 10 s are not analyzed due to occasional, small voids immediately at the time of mouse placement within the enclosure. For analysis, since micturition behavior was more variable than gait (many mice had urinary frequency in only half of the MVT tests on Weeks 5–10), we averaged the number of voids across Weeks 5–10.

### Open field test and novel object recognition

First, mice were placed into an open field arena (open-top, 40 × 40 × 40 cm) and recorded with a down-facing camera for 10 min. This open field test also served as habituation for the subsequent novel object recognition test in the same arena. Typically, two mice were recorded at a time, using side-by-side arenas and two, separate cameras. Mice were then returned to their home cages for roughly 3 min, while the apparatus was cleaned with ethanol. Two identical objects were placed in the midline of the arena, equidistant each side, leaving enough space for the mouse to explore each object on all sides. The mice were then returned to the apparatus and recorded for another 10 min. The mice were again returned to their home cage for roughly 3 additional minutes while the apparatus was cleaned, and new objects were placed in the same two locations; one was identical to the previous objects, and the other (the novel object) was a different shape and color. The side of the arena with the novel object was randomized. The mouse was returned to the arena for another 10 min recording.

Open field test videos were later analyzed in EthoVision (Noldus) to measure total distance moved and time spent moving. Average velocity while moving was calculated as the quotient of these two measured values. Novel object recognition videos were analyzed manually to measure the time spent directly interacting with each object, which we defined as facing the object, in close proximity to it (<2 cm), typically including sniffing and sometimes including rearing and touching the object with the paws. Beginning at the time of first object interaction, 3 min were scored. Total time investigating each object was calculated. To assess novel object recognition and memory, we calculated the discrimination index for each mouse (*t_n_* − *t_f_*) / (*t_n_* + *t_f_*) where *t_n_* is the time with the novel object and *t_f_* is the time with the familiar object. We then compared this to chance, which would be 0 (equal time with both objects). As a positive control for task engagement, we also summed the total time spent interacting with both objects for each mouse.

### Barnes maze

For this test, we used an elevated circular platform 122 cm in diameter with 40 escape holes (diameter 5 cm) spaced evenly around the perimeter and 5 cm from the edge (Barnes, 1979). Thirty of the escape holes were permanently blocked, leaving 10 evenly spaced holes available. For every test, one escape hole was left open, with an open escape chamber beneath it, while the other nine holes contained a perforated disk blocking the entrance to identical escape chambers. Each escape chamber was held by two rails beneath the platform surface. Crescent-shaped caps placed atop the medial edge of all 10 holes prevented mice from seeing which hole was open. At the start of each trial, mice were placed under a large, inverted funnel in the center of the apparatus. After 5 s, the funnel was removed, allowing the mouse to explore. Once the mouse entered the chamber through the open escape hole, a tunnel was attached to the chamber, providing direct access to the home cage.

For the first trial of Day 1 only, dirty bedding from the home cage was placed in the chamber beneath the open escape hole as an additional, olfactory incentive for the mouse. This chamber was subsequently replaced with a new, clean chamber. On Day 1, six trials were conducted with the same location of the open escape hole relative to surrounding visual cues in the room, but the table and associated chambers were rotated before each trial to ensure that the mouse could not simply use scent cues to find the open hole. On consecutive Days 2 and 3, five tests with the original escape hole location from Day 1 were performed. All tests were recorded with an overhead camera, and videos were analyzed using ANY-maze (Stoelting) to measure latency to escape.

### Prepulse inhibition

Mice were placed in individual noise isolation chambers in an SR-LAB startle-response system (SD Instruments) with lights on. Before testing all mice, each chamber was calibrated using a decibel meter and oscillating mass standard according to the manufacturer's specifications. After habituation for 10 min to a background tone slightly louder than ambient noise, each mouse underwent eight trials with a 120 dB tone and 15 s intertrial interval. This was followed by 60 mixed, randomized trials with one of five prepulse stimuli (no tone; 120 dB tone with no prepulse; 5 dB prepulse tone followed by 120 dB tone, 10 dB prepulse followed by 120 dB tone, 15 dB prepulse followed by 120 dB tone). Movement in response to the 120 dB tone (or none, in the no-tone trial) was measured by an accelerometer (mV) and recorded in SR-LAB. Analyzed results were exported to Excel, where we calculated average response for each mouse to each stimulus type.

### Auditory brainstem response

Click-evoked auditory brainstem responses (ABRs) were recorded as described previously (Yoshimura et al., 2019). Mice were anesthetized with ketamine–xylazine (100 and 10 mg/kg, i.p.). Recordings were conducted from both ears of all animals on a heating pad using electrodes placed subcutaneously in the vertex and underneath the left or right ear. Clicks were square-wave 100 ms pulses. Tone bursts were 3 ms in length at distinct 8, 16, 24, and 32 kHz frequencies. ABRs were measured with BioSigRZ (Tucker-Davis Technologies), adjusting the sound pressure levels between 20 and 100 dB, in 5 dB increments, in both ears. Electrical signals were averaged over 512 repetitions. We compared both ABR latency to wave peak and ABR threshold, defined as the lowest sound level at which a reproducible waveform could be observed.

### Sucrose preference testing

Mice were individually housed and the water spigot previously providing *ad libitum* access was removed. In its place, each mouse received a 30 ml bottle of fresh, distilled water for 48 h prior to testing. *Ad libitum* food access continued throughout this experiment. For the following 24 h, each mouse was given one bottle of fresh, distilled water plus one bottle of 1% sucrose (dissolved in fresh distilled water). Each full bottle was weighed immediately before placing it in the cage. The cage side with sucrose (right or left) was randomized. After 24 h, all bottles were weighed again. The final weight was subtracted from the original weight for each bottle, and the percent sucrose relative to total intake [sucrose / (sucrose + water)] was calculated for each mouse.

### Food and water intake, body weight, and pair feeding

We evaluated the initial, perioperative food intake and body weight in a cohort of *n* = 7 kaolin-injected and *n* = 7 saline-injected mice. Beginning 3 d prior to surgery and for 21–30 d following surgery, mice were individually housed atop grid cage bottoms with *ad libitum* access to food and water. Every 3 d, all food in the food hopper was weighed, as well as any crumbs that had fallen through the grid floor (after sifting and removing feces). This amount was then subtracted from the previous food weight to calculate total food consumption over the previous 3 d. Mouse body weight was also measured every 3 d.

A separate, pair feeding (also known as yoked-control) experiment followed largely the same protocol, except that saline-injected control mice (*n* = 6) and uninjected control mice (*n* = 6) were paired 1:1:1 with each kaolin-injected mouse (*n* = 6). Pair-fed mice were 3 d delayed in their protocol timeline and were given the same amount of food (+20%, to allow for crumbs falling through the grid cage-bottom) that hydrocephalic mice had consumed over the previous 3 d.

To further break down the time course of changes in food consumption, and to measure water consumption, mice were placed in a BioDAQ system (Research Diets). They were individually housed in this system 5 d before surgery. After habituating for 2 d, we began continuous recordings of food and water intake for 3 preoperative days and after returning mice immediately after recovery from anesthesia, for an additional 14 postoperative days. Each cage in this system is attached to two scales that continuously weigh (±10 mg) two respective hoppers for that cage. We calibrated all BioDAQ scales according to the manufacturer's instructions before recording, and we left the DataViewer bout filter at the standard setting of −0.05 g. For each cage, one hopper contained TD 96208 chow (Envigo). The other hopper held a bottle containing distilled water. We used ALPHA-dri+ PLUS (Shepherd) bedding because other bedding material is sometimes pushed through the hopper onto the scale. We exported data from the DataViewer recording software (Research Diets) and calculated total consumption per day in Microsoft Excel.

### Perfusions and histology

Mice were anesthetized with a mixture of ketamine–xylazine (150–15 mg/kg, i.p., dissolved in sterile 0.9% saline) and then perfused transcardially with phosphate-buffered saline (PBS), followed by 10% formalin-PBS (SF100-20, Fisher Scientific). After perfusion, brains were removed and fixed overnight in 10% formalin-PBS. We sectioned each brain into 40-µm-thick coronal slices using a freezing microtome and collected tissue sections into separate, 1-in-3 series. Sections were stored in cryoprotectant solution at −20°C until further processing. For immunofluorescence labeling, we removed tissue sections from cryoprotectant and rinsed them in PBS before loading them into a primary antibody solution. Chicken-anti-GFAP (glial fibrillary acidic protein; Millipore AB5541, diluted 1:2,000, or Aves “GFAP,” diluted 1:1,000) was added to a PBS solution containing 0.25% Triton X-100 (BP151-500, Fisher), 2% normal donkey serum (NDS, 017-000-121, Jackson ImmunoResearch), and 0.05% sodium azide (14314, Alfa Aesar) as a preservative (PBT-NDS-azide). We incubated these sections overnight at room temperature on a tissue shaker. The following morning, the sections were washed three times in PBS and then incubated for 2 h at room temperature in PBT-NDS-azide solution containing a species-specific donkey secondary antibody conjugated to either Alexa Fluor 488 or Cy3 (Jackson ImmunoResearch #703-545-155, 703-165-155; diluted 1:500 or 1:1,000, respectively). These sections were then washed three times in PBS, mounted on glass slides (#2575-plus; Brain Research Laboratories), and coverslipped using Vectashield with DAPI (VectorLabs). Slides were stored in slide folders at 4°C until imaging.

For whole-skull hematoxylin and eosin (H&E) staining, mice were perfused as above; then muscle, fat, eyes, and skin tissue were removed from the skull; and the entire skull was placed in 10% formalin for 7 d. Following fixation, for decalcification they were placed in EDTA and rotated continuously for 3–7 d, replacing the EDTA solution daily. Decalcification was checked with a 1:1:1 mixture of used EDTA, 5% ammonium hydroxide, and 5% ammonium oxalate. Skulls were then placed inside a Sakura Tissue-Tek VIP 6 AI, and a program for brain paraffin embedding was run, where the heads were progressed through solutions of increasing ethanol concentrations (70–100%, then 100% repeated three times) for 45 min each, then a pro-par solution three times for 1 h each, and finally paraffin (Leica Biosystems, EM-400) four times (20–40 min). We then embedded the skulls in a paraffin block prior to sectioning with a Leica Biocut paraffin microtome. Ten-micrometer-thick parasagittal sections were cut, and 1-in-15 sections were collected and immediately mounted onto glass slides. These slides were later placed in a warmer (58°C) for 15–30 min to remove paraffin from the tissue sections. The slides were then placed into an autostainer and run on an H&E protocol, where the slides were dipped in xylenes three times for 3 min each, then decreasing concentrations of ethanol (100–70%) for 1–3 min, before dipping in hematoxylin for 6 min, washing, and dipping in eosin for 30 s. After staining, they were dipped in increasing ethanol concentrations and then xylenes three times for 1 min each. Slides were kept in xylenes until coverslipping with S-Mounting Medium (Newcomer Supply #6751).

For scanning electron microscopy, mice were perfused as above but with freshly prepared 4% paraformaldehyde EM grade. Following perfusion, the brain was removed and immediately placed in half-strength Karnovsky's fixative in 0.1 M Na cacodylate, pH 7.2, and postfixed, protected from light, for 72 h at +4°C. Briefly, the tissues were postfixed with an aqueous solution of 1% osmium tetroxide solution in 0.1 M Na cacodylate, pH 7.2, and followed by dehydration in a graded ethanol series up to 100%. The samples were then dried using the critical point drying of CO2, mounted on aluminum SEM pins using colloidal silver, and sputter coated with gold and palladium particles using an Emitech Sputter Coater K550.

### Scanning electron microscopy

Forebrain and brainstem samples were imaged using a scanning electron microscope (Hitachi S-4800) with an acceleration voltage of 3 kV and an emission current of 10 µA. Magnification ranged from 2,000× to 4,000× and working distance from 9,700 to 10,900 μm.

### Light microscopy

All slides were imaged using a VS120 whole-slide scanning microscope (Olympus). We began by first acquiring a 2× overview scan then using a 10× objective to scan all tissue sections. After reviewing data in OlyVIA (Olympus), we used cellSens (Olympus) to crop full-resolution images and Adobe Photoshop to adjust brightness and contrast. We used Adobe Illustrator to arrange images and add lettering for final figure layouts. Scale bars were traced in Illustrator atop calibrated lines from cellSens to produce clean white or black line in each panel.

For GFAP immunofluorescence analysis, all brains were scanned at the same exposure time (75 ms) using the same 10× object. The image upper threshold in VS-ASW (Olympus) was set to 40,000 in all cases. Three evenly spaced sections through the caudal cerebral aqueduct (∼360 µm interval) were chosen from each brain (spanning the mid-aqueduct through the level at which the aqueduct expands into the fourth ventricle). An ROI that extended 500 µm in all directions from the ventricle wall was then exported as a TIFF, which was opened in ImageJ to calculate integrated intensity (the mean gray value of all pixels in the image). This result was averaged across the three rostrocaudal ROIs in each case.

### Data analysis and statistics

We used Microsoft Excel to organize and preview datasets. We used GraphPad Prism 9.4.1 to plot results for figures and to run statistical analyses. For experiments in which two different groups were compared, we used Student's *t* test. For comparisons of more than two groups, we used a one-way ANOVA, with Tukey's multiple-comparisons correction. For comparisons of two groups across time, we used a mixed effects model (REML, different group sizes) or two-way RM ANOVA (same group sizes) with Sidak's multiple-comparisons correction. Each is detailed in the figure legends. Numerical results are presented as mean ± standard deviation, except for the Barnes maze time-course graph, which displays standard error of the mean.

Retrospective post hoc analysis showed that there was no difference in age or weight between control and experimental mice on most experiments. The only exception was a small difference in the average age of the female mice in the prepulse inhibition experiment (where no significant experimental differences were found, as shown below). Additionally, there were no sex differences on retrospective comparisons of most experiments. The only exceptions were the open field test and ABR test, where results are shown separately.

### Study approval

All experiments were conducted in accordance with the guidelines of the Institutional Animal Care and Use Committee at the University of Iowa.

## Results

### Inducing hydrocephalus

**Figure 1.**
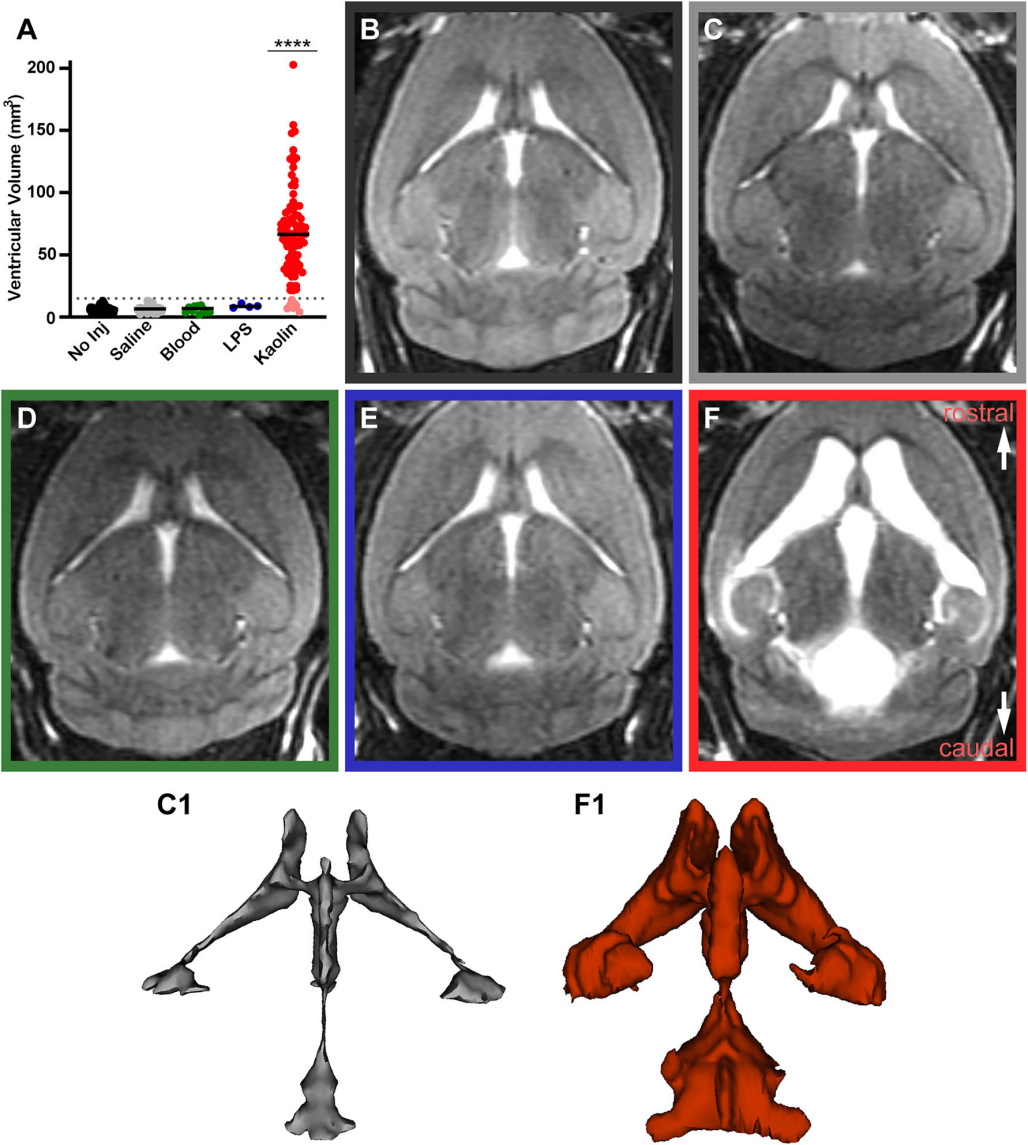
Cisternal delivery of kaolin reliably produced hydrocephalus. ***A***, Ventricular volumes (mm^3^) of mice prior to surgery (No Inj) and after injection of saline (*n* = 52), blood (*n* = 11), lipopolysaccharide (LPS, *n* = 4), or kaolin (*n* = 90) into the cisterna magna (***p < 0.0001 kaolin vs every other condition, one-way ANOVA with Tukey's multiple-comparisons correction). ***B–F***, Representative brain magnetic resonance imaging (MRI, horizontal plane) prior to surgery (***B***) and after injection with saline (***C***), autologous blood (***D***), LPS (***E***), or kaolin (***F***) into the cisterna magna. ***C1***, ***F1***, 3D rendering of cerebral ventricles from the saline-injected and kaolin-injected mice shown above (***C***, ***F***).

We began by measuring the total cerebral ventricular volume in unoperated adult mice (*n* = 60). Preoperative ventricular volumes were uniformly small (*n* = 60; range, 1.91–13.01 mm^3^; mean, 6.51 ± 2.39 mm^3^). No mice had spontaneous hydrocephalus (Fig. 1*A*,*B*). Using this baseline information, we defined hydrocephalus for all remaining experiments as a total cerebral ventricular volume >14 mm^3^, which is 3 standard deviations above the mean of unoperated mice.

Next, we hypothesized that blood in the subarachnoid space would induce hydrocephalus in mice because a well-known risk factor for human NPH is prior subarachnoid hemorrhage in the basilar cisterns (Bagley, 1928; Germanwala et al., 2010; Daou et al., 2016). However, delivering increasing amounts (1–10 µl) of autologous blood into the subarachnoid space via the cisterna magna did not induce hydrocephalus in any mice (*n* = 11; ventricular volumes, 1.81–9.54 mm^3^ at 6–7 weeks postoperative; mean, 6.10 ± 2.64 mm^3^; Fig. 1*D*). Another established risk factor for NPH is bacterial meningitis, so we next attempted to induce hydrocephalus using the bacterial endotoxin lipopolysaccharide (LPS). However, LPS injections into the cisterna magna did not induce hydrocephalus in any mice (*n* = 4; ventricular volumes, 7.51–11.01 mm^3^ at 6–7 weeks postoperative; mean, 8.86 ± 1.52 mm^3^; Fig. 1*E*).

Less well-known risk factors for NPH include the disease neurosarcoidosis, which produces granulomatous neuropathology (Gupta et al., 1994; Berntsson et al., 2023). To induce this form of inflammation, we used kaolin (aluminum silicate), which produces granulomatous inflammation in peripheral tissues (Morgan et al., 1988; Burt et al., 2021) and induces hydrocephalus in various animal species (Hochwald, 1985; Di Curzio, 2018; Chen et al., 2023). Injecting kaolin into the cisterna magna caused ventriculomegaly (4.06–202.75 mm^3^; mean, 66.91 ± 37.32 mm^3^; *n* = 90; Fig. 1*F*), with 91% of kaolin-injected mice developing hydrocephalus. In contrast, saline-injected control mice never developed hydrocephalus (2.17–13.07 mm^3^; mean, 6.75 ± 2.49 mm^3^; *n* = 52; Fig. 1*C*). This difference is clearly visible on 3D renderings of the cerebral ventricles (Movie 1; Fig. 1*C1* vs *F1*). Cerebral ventricular volumes of kaolin-injected mice were significantly different from all other groups (*p* < 0.0001; Fig. 1*A*).

**Figure 2.**
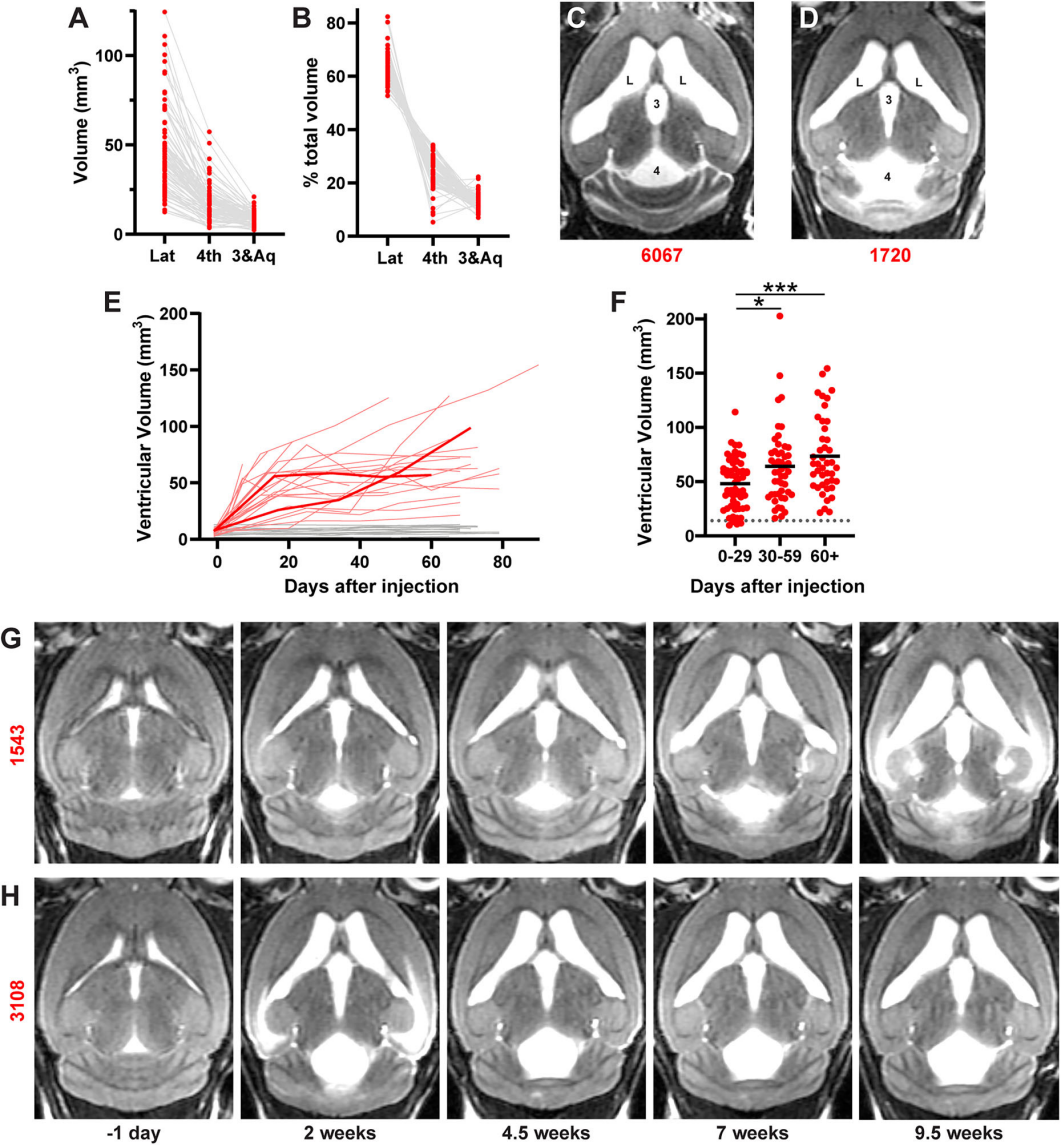
Hydrocephalus developed progressively with variable patterns of ventriculomegaly. ***A***, Total volume of separate ventricles for each hydrocephalic mouse (*n* = 82). ***B***, Percentage of total ventricular volume for each hydrocephalic mouse (*n* = 82). ***C***, Some mice had large lateral ventricles and a relatively smaller fourth ventricle. ***D***, Others had an enlarged fourth ventricle and relatively smaller lateral ventricles. ***E***, Serial brain MRIs reveal stable ventricular volume in saline-injected control mice and variable, progressive increases after kaolin injection. Bold red lines represent mice 1,543 (***G***) and 3,108 (***H***; *n* = 41). ***F***, Comparison of ventricular volumes all MRIs binned into different time periods in which they were obtained [*p* = 0.003; *p* = 0.024, 0–29 d (*n* = 57) vs 30–59 d (*n* = 49); *p* = 0.0002 0–29 d vs 60+ d (*n* = 30); *p* = 0.32 30–59 d vs 60+ d by one-way ANOVA with Tukey's multiple-comparisons correction]. ***G***, Serial MRIs of representative mouse 1,543, with gradually enlarging ventricles. ***H***, Serial MRIs of representative mouse 3,108 with earlier ventricular expansion that leveled off after 2 weeks. Extended data on ventriculomegaly are shown in Extended Data Figure 2-1 and Movie 1.

Kaolin induced varying patterns of ventricular enlargement. In some mice, the lateral ventricular expansion was vast (Fig. 2*C*). In others, the lateral ventricles were less expanded, and the cerebral aqueduct and fourth ventricle were extremely enlarged (Fig. 2*D*). The lateral ventricles ranged from 53 to 83% of total ventricular volume, and the fourth ventricle ranged more widely, from 5 up to 34% (Fig. 2*A*,*B*).

10.1523/ENEURO.0412-24.2024.f2-1 Figure 2-1 Total ventricular volumes measured 6-10 weeks after injecting 15% (n = 75) vs. 12.5% (n = 6) vs. 10% (n = 9) kaolin into the cisterna magna (p = 0.00835 One-way ANOVA, 15% vs 10% p = 0.012 with Tukey’s multiple comparisons correction). Download Figure 2-1, TIF file.

Hydrocephalus developed gradually after kaolin injection, primarily in the first few weeks and more slowly thereafter (Fig. 2*E*). In mice that developed hydrocephalus, cerebral ventricular volumes became larger later in the experiment than in the first 30 d following kaolin injection (*p* = 0.0002; Fig. 2*F*). Ventriculomegaly continued progressing in some, while others stabilized as early as 2 weeks after injection (Fig. 2*G*,*H*).

In addition to variability in rate of expansion, we also found variability in final ventricular volume. The smallest total ventricular volume in the hydrocephalic group was 16.89 mm^3^ and the largest was 202.75 mm^3^. We also found that the highest kaolin concentration we tested (15%) produced hydrocephalus more reliably than the lowest (10%; *p* = 0.012; Extended Data Fig. 2-1).

### Kaolin induces communicating hydrocephalus with normal intracranial pressure

**Figure 3.**
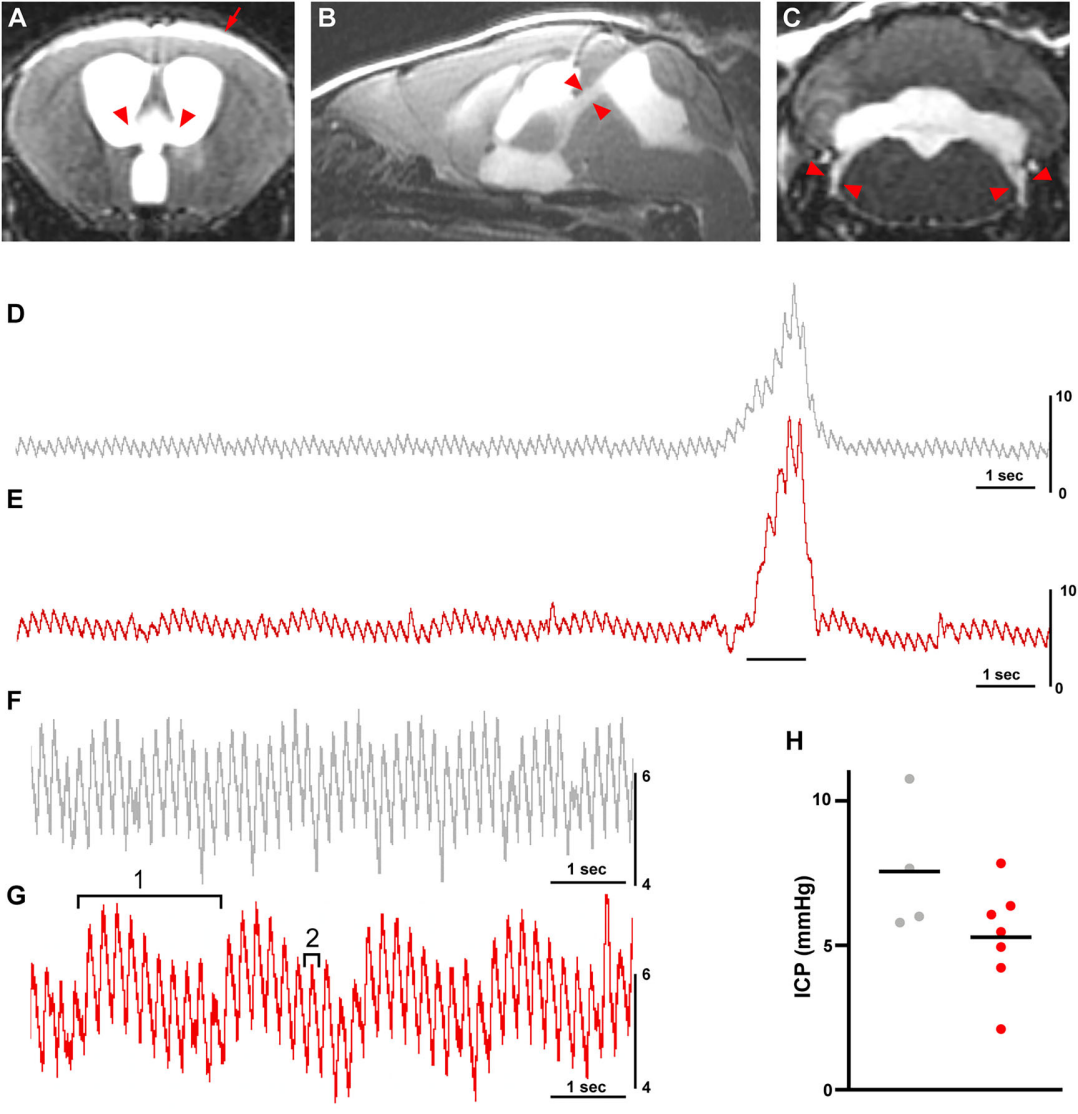
Kaolin produces communicating (nonobstructive) hydrocephalus without increased intracranial pressure. ***A***, Coronal MRI showing open foramina of Monro (red arrowheads) and expanded subarachnoid space (red arrow). ***B***, Sagittal MRI showing open cerebral aqueduct (red arrowheads). ***C***, Coronal MRI showing open lateral foramina of Luschka (red arrowheads). ***D***, ***E***, Intracranial pressure (ICP) recordings from saline-injected control (***D***) and kaolin-injected hydrocephalic (***E***) mice. Black bar represents gentle abdominal squeeze. ***F***, ***G***, Zoomed-in ICP trace showing respiratory (1) and cardiac (2) component waveforms in control (***F***) and hydrocephalic (***G***) mice. ***H***, Average 10 s ICP of saline-injected control (gray, *n* = 4) and kaolin-injected hydrocephalic (red, *n* = 7) mice were not different (*p* = 0.10 by Student's *t* test). Extended data are shown in Extended Data Figure 3-1.

MRI analysis confirmed that kaolin-induced ventriculomegaly is a form of communicating hydrocephalus. The foramina of Monro were patent in all hydrocephalic mice (*n* = 82; Fig. 3*A*). In all but three, we were able to definitively identify a patent cerebral aqueduct (Fig. 3*B*), which was undetectable (below the limit of resolution) in all saline-injected mice (*n* = 52). Additionally, both lateral foramina of Luschka, through which CSF exits the fourth ventricle to reach the basilar cisterns, were visibly patent in at least 85% of hydrocephalic mice (*n* = 70/82; Fig. 3*C*). In contrast, the presumably patent foramina of Luschka were below the MRI limit of detection in most saline-injected mice (77%; *n* = 40 of 52). In addition to ventriculomegaly, some mice injected with kaolin (*n* = 19/82) had an enlarged subarachnoid CSF space dorsal to the cerebral hemispheres (Fig. 3*A*), reminiscent of DESH (disproportionately enlarged subarachnoid space in hydrocephalus) reported in some NPH patients (Hashimoto et al., 2010). This MRI finding was not present in any saline-injected mice.

10.1523/ENEURO.0412-24.2024.f3-1 Figure 3-1 **(A, B)** Dorsal (top) and ventral (bottom) images of two representative brains in which Evans Blue dye was injected into the lateral ventricle 4 weeks after kaolin injection. **(C)** ICP measurements at different timepoints following kaolin injection (p = 0.68, one-way ANOVA of hydrocephalic groups (n = 4 each) across different time points; p = 0.37 t-test of saline vs. kaolin at week 9, n = 3 saline, n = 4 kaolin). **(D–E)** Average ICP waveform amplitudes (n = 4 saline, 7 kaolin) of **(D)** Respiratory (slow) waves (p = 0.57, t-test) and **(E)** Cardiac (fast) waves (p = 0.85, t-test). Download Figure 3-1, TIF file.

As a further test of interventricular patency, we injected Evans blue dye into the right lateral ventricle under anesthesia (*n* = 5 hydrocephalic mice). Dye appeared in the cisterna magna within minutes, coating the basilar cisterns and periarterial spaces around the brain by the time the mice were perfused the same day (Extended Data Fig. 3-1).

A defining feature of NPH is the lack of elevated intracranial pressure (ICP). We measured ICP at 1, 2, 3, and 9 weeks (*n* = 4 mice/week) and found no difference between weeks (*p* = 0.52; Extended Data Fig. 3-1*C*). Nor was there an elevation in kaolin-injected mice relative to saline-injected control mice at 9 weeks (*p* = 0.37; *n* = 3; Extended Data Fig. 3-1*C*). These initial recordings did not resolve ICP waveform morphology, so we used a more sensitive pressure sensor (Millar SPR-671) to perform additional recordings in hydrocephalic and control mice at 11 weeks. This more sensitive transducer revealed fine ICP waveform morphology, and pressing on the abdomen caused a sharp, transient increase in pressure in each mouse (examples shown in Fig. 3*D*,*E*). Cardiac waveforms (5–7 Hz) were seen in all mice, ranging 1.3–1.9 mmHg from peak to trough. Most mice also had respiratory waveforms (∼1 Hz), ranging 1.5–4.5 mmHg from peak to trough (Fig. 3*F*,*G*). There were no significant differences in the amplitude of cardiac (*p* = 0.85) or respiratory (*p* = 0.57) waveforms between hydrocephalic and control mice (Extended Data Fig. 3-1*D,E*). Once again, ICP was not elevated in kaolin-injected mice (*n* = 7; 5.27 ± 1.80 mmHg) relative to saline-injected control mice (*n* = 4; 7.53 ± 2.29 mmHg; *p* = 0.10, Fig. 3*H*).

### Hydrocephalic mice have a slow and unsteady gait

While every vehicle-injected mouse had a normal-appearing gait, all hydrocephalic mice appeared slightly unsteady. To assess their apparent deficit, we used a variety of gait and balance tests.

**Figure 4.**
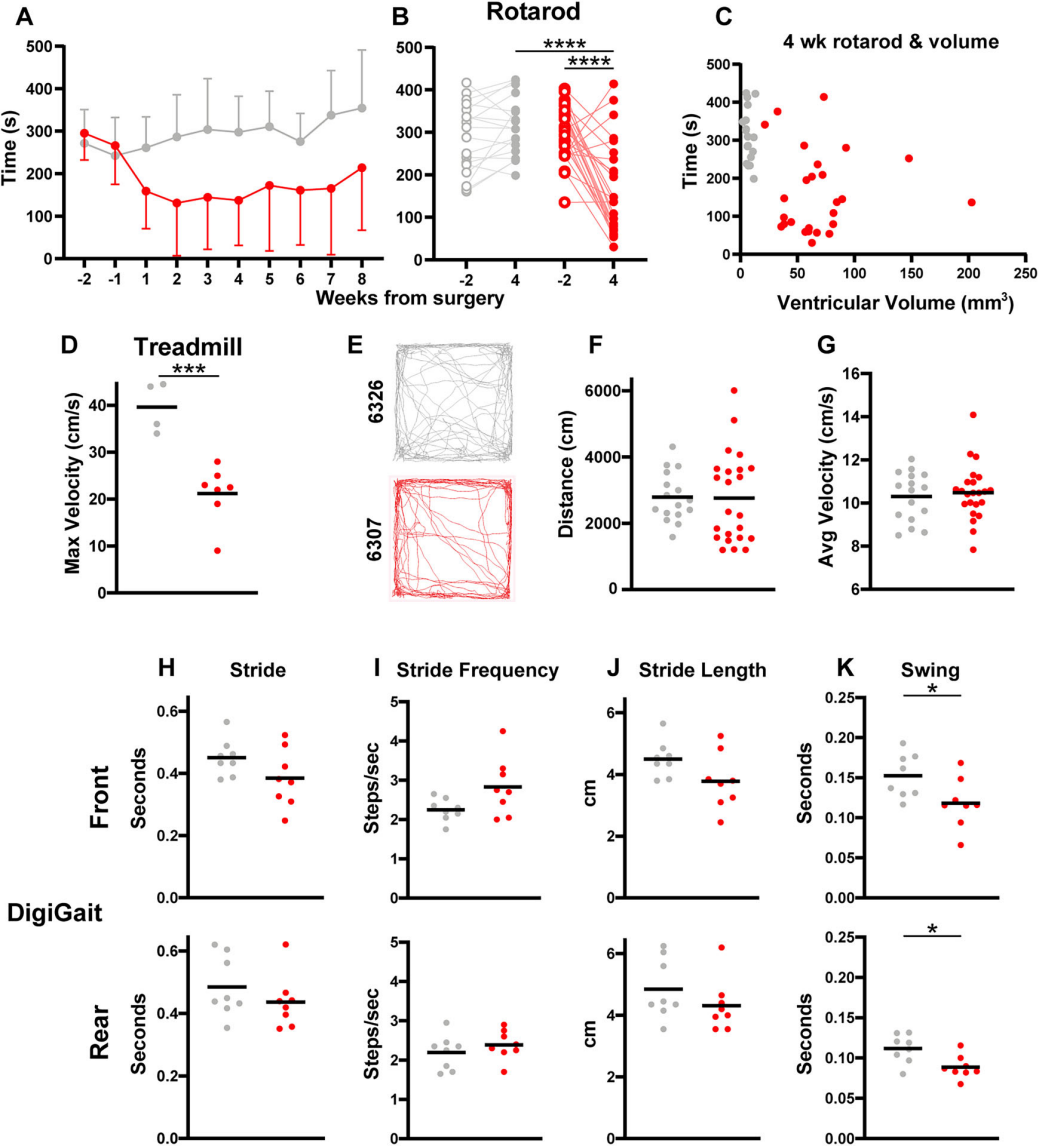
Hydrocephalic mice have a slow and unsteady gait. For all graphs, red represents kaolin-injected hydrocephalic mice and gray represents saline-injected control mice. ***A***, Average weekly time on rotarod from 2 weeks before to 8 weeks after surgery (*n* = 14 kaolin; *n* = 10 saline). ***B***, Length of time each individual mouse lasted on the rotarod at 2 weeks prior and 4 weeks following surgery (****p < 0.0001; *p* = 0.97 for saline −2 vs kaolin −2, by one-way ANOVA with Tukey's multiple comparisons; *n* = 26 kaolin; *n* = 18 saline). ***C***, Rotarod performance versus cerebroventricular volume on final MRI (*p* = 0.61; *n* = 26 kaolin; *n* = 18 saline). ***D***, Maximum speed on an accelerating treadmill (***p = 0.0007 by *t* test; *n* = 7 kaolin; *n* = 4 saline). ***E***, Tracing of movement for representative control mouse and hydrocephalic mouse during a 10 min open field test (*n* = 23 kaolin; *n* = 16 saline). ***F***, Distance moved during a 10 min open field test (*p* = 0.95 by *t* test; *n* = 23 kaolin; *n* = 16 saline). ***G***, Average velocity while moving on a 10 min open field test (*p* = 0.66 by *t* test; *n* = 8 kaolin; *n* = 8 saline). ***H–K***, DigiGait data with forepaws on top row and hindpaws on bottom row, all *p* values from Student's *t* test. ***H***, Average stride time in seconds (forepaws *p* = 0.11; hindpaws *p* = 0.31). ***I***, Average stride frequency in steps/second (forepaws *p* = 0.056; hindpaws *p* = 0.36). ***J***, Average stride length in cm (forepaws *p* = 0.082; hindpaws *p* = 0.27). ***K***, Average swing time in seconds (* forepaws *p* = 0.036; * hindpaws *p* = 0.012). Hydrocephalic and control gait examples are shown in Movie 2.

On the rotarod test, hydrocephalic male mice performed worse than control mice. While both groups performed similarly prior to surgery, a performance deficit appeared early in hydrocephalic mice and continued for many weeks after kaolin injection (Fig. 4*A*). At 4 weeks, hydrocephalic mice performed worse than saline-injected controls (*n* = 26 hydrocephalic; *n* = 18 controls; 161.94 ± 108.70 vs 312.51 ± 69.46 s; *p* < 0.0001; Fig. 4*B*). Control mice had no change in performance from before to after saline injection, but hydrocephalic mice performed significantly worse 4 weeks after than 2 weeks prior to kaolin injection (298.43 ± 64.74 s vs 161.94 ± 108.70; *p* < 0.0001; Fig. 4*B*). Surprisingly, rotarod performance did not correlate with overall ventricular volume (Fig. 4*C*).

Despite the visible unsteadiness and poor rotarod performance of hydrocephalic mice, overall locomotion was not grossly impaired. In an open field arena, they covered a similar distance (*n* = 23 hydrocephalic; *n* = 16 controls; 2,767 ± 1,354 vs 2,791 ± 745 cm; *p* = 0.96; Fig. 4*F*) and had a similar average velocity while moving as controls (10.48 ± 1.27 vs 10.31 ± 1.14; *p* = 0.67; Fig. 4*G*).

However, hydrocephalic mice did not move as fast on a treadmill. Four weeks after surgery, their maximum gait speed was significantly less than that of control mice (*n* = 7 hydrocephalic 21.21 ± 6.05 cm/s vs *n* = 4 controls 39.63 ± 5.40 cm/s; *p* = 0.0007; Fig. 4*D*). Representative gaits of control and hydrocephalic mice are shown in Movie 2. Formal gait analysis indicated that hydrocephalic mice had significantly less swing time in both the front (*n* = 8 hydrocephalic; *n* = 8 control; 0.12 ± 0.03 vs 0.15 ± 0.03 s; *p* = 0.036) and the rear paws (0.09 ± 0.01 vs 0.11 ± 0.02 s; *p* = 0.12). However, the stride length, stride frequency, and overall stride time was not different between hydrocephalic mice and controls (Fig. 4*H–K*).

### Hydrocephalic mice develop urinary frequency and become incontinent

**Figure 5.**
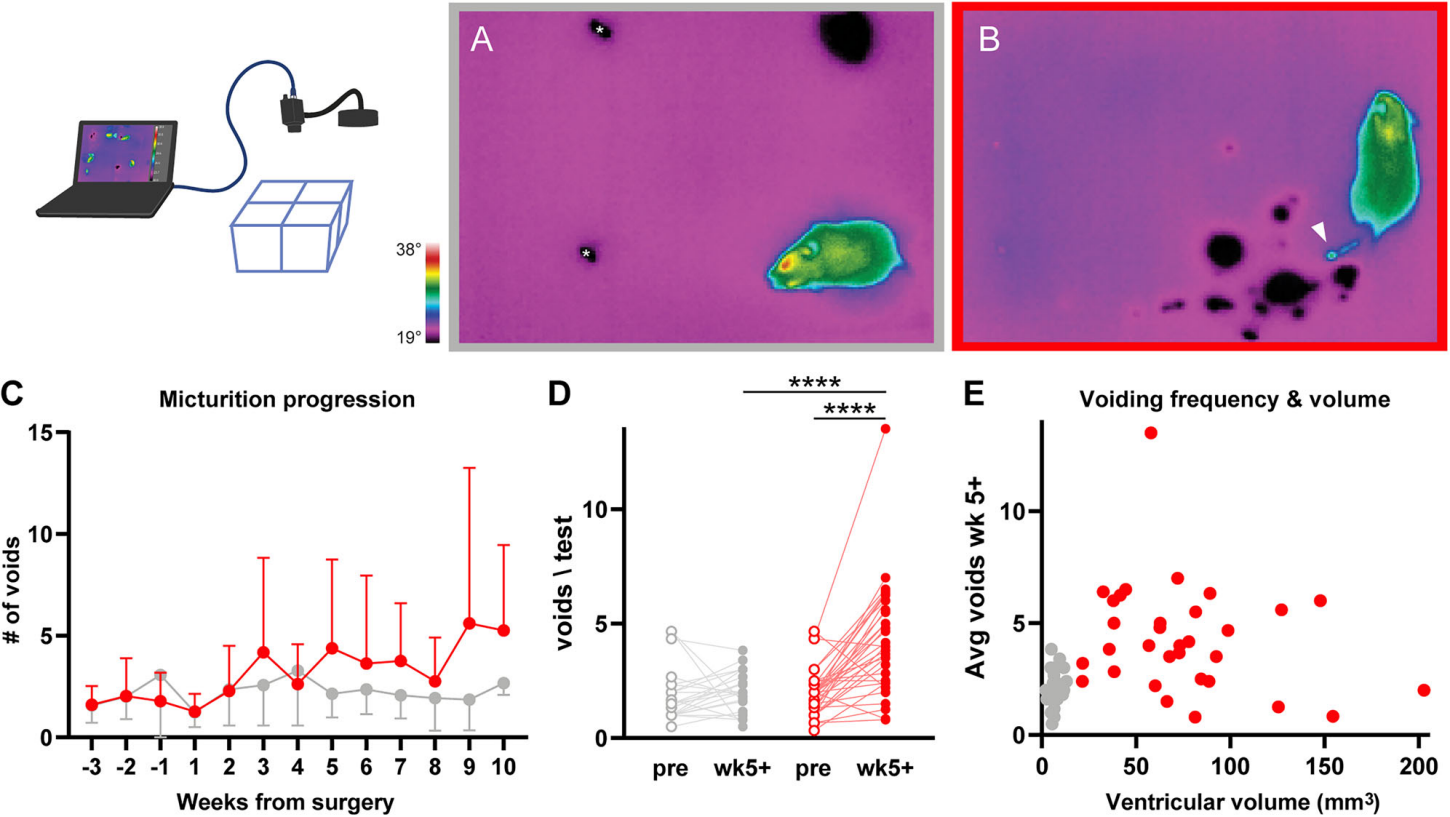
Hydrocephalic mice developed urinary frequency. Graphic represents experimental setup for micturition video thermography (MVT) recordings. ***A***, Frame from MVT video of a control mouse with one void in the top right (asterisks denote feces). ***B***, Frame from MVT video of hydrocephalic mouse with urinary incontinence with small voids clustered in the bottom-right and a fresh, small void (white arrowhead) that appeared while the mouse was walking. See Movie 3 for details. ***C***, Urinary frequency appeared several weeks after surgery in most mice and increased over time (*n* = 20 kaolin; *n* = 14 saline). ***D***, Average number of voids per MVT session in presurgery tests and in Weeks 5–10 postsurgery [****p < 0.0001 kaolin (*n* = 32) vs saline-injected (*n* = 22) by one-way ANOVA with Tukey's multiple-comparisons correction]. ***E***, Average number of voids per MVT test (Weeks 5–10) compared with ventricular volume on MRI (*p* = 0.18; *n* = 32 kaolin; *n* = 22 saline). Examples of voiding behavior are shown in Movie 3.

To assess urinary frequency and continence, we developed a novel, noninvasive assay called micturition video thermography (MVT; Fig. 5; Verstegen et al., 2020). This assay revealed that hydrocephalic mice voided more frequently, and this phenotype developed 4–5 weeks after kaolin injection (Fig. 5*C*). Analyzing weeks 5–10, hydrocephalic mice (*n* = 32; 4.28 ± 2.45 voids) voided more frequently than they did in the 3 weeks before kaolin injection (1.99 ± 1.03) and more frequently than vehicle-injected control mice (*n* = 22; 2.04 ± 0.89; *p* < 0.0001; Fig. 5*D*). Prior to surgery, all mice voided an average of 1.9 ± 1.0 times in 2 h (Fig. 5*E*), similar to our previous results in C57BL/6J mice (Verstegen et al., 2020). We used this baseline information to define urinary frequency as five or more voids in a 2 h MVT test, which is 3 standard deviations above the mean. Using this definition, among the 37 hydrocephalic mice tested (*n* = 25 male; *n* = 12 female), 24 exhibited urinary frequency in at least one test (65%; Fig. 5*E*), and 18 were frequent in two or more tests (49%). Only two of these 18 animals had been frequent in any presurgery test. In contrast, of the 22 vehicle-injected control mice, four became frequent on a postsurgery test (18%), only one was frequent on more than a single test, and three of these four mice had been frequent in a presurgery test.

Immediately before voiding, mice typically exhibit continent behavior by backing into a corner, raising the tail, and pausing (Verstegen et al., 2020). In contrast, 60% of hydrocephalic mice exhibited incontinent voiding behavior in at least one test (Movie 3). Instead of normal voiding behavior, they voided while walking, without raising the tail or pausing (16% of all postsurgery voids in hydrocephalic mice, vs none in control mice). Additionally, incontinent voids were small (below 0.5 cm in diameter, <5 µl) and were scattered across the floor instead of confined to the corners or edges of the enclosure. After surgery, 72% (715/987) of hydrocephalic voids were in the corners or along edges, compared with 88% (330/374) in saline-injected mice and 87% (240/275) in presurgical tests. Incontinence was rare before the fifth week, but most mice with incontinent voids on any one test also exhibited incontinent behavior on one or two additional tests.

### Learning and memory in hydrocephalic mice

**Figure 6.**
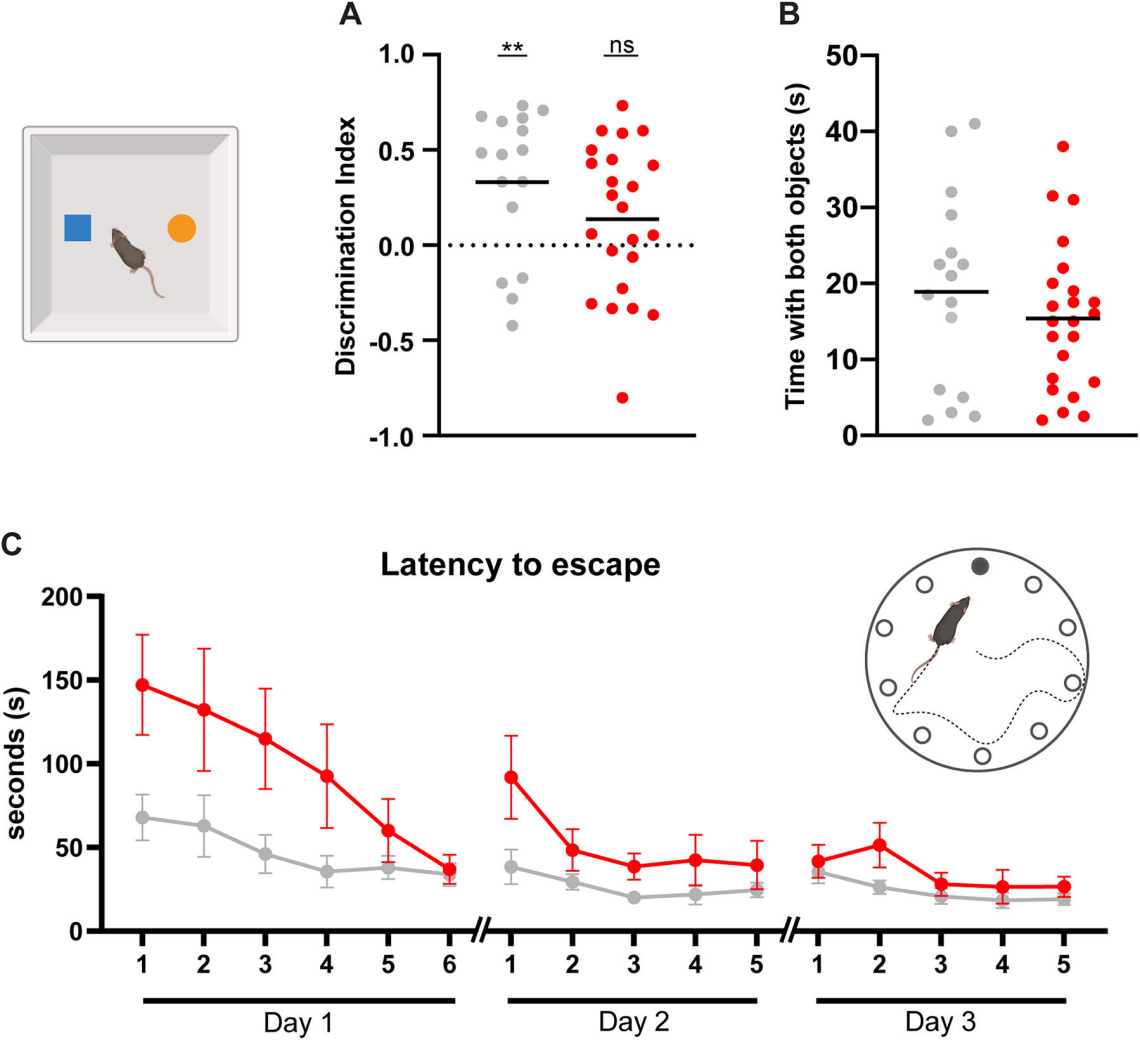
Hydrocephalic mice had a mild learning impairment. On the novel object recognition test, (***A***) the discrimination index (DI) of saline-injected control mice (gray, *n* = 16) was significantly different from chance (**p = 0.004), but the DI of kaolin-injected hydrocephalic (red, *n* = 23) mice was not (*p* = 0.12). These two groups of mice were not significantly different (*p* = 0.14 by Student's two-tailed *t* test). ***B***, Control and hydrocephalic mice spent a similar amount of total time interacting with the objects (*p* = 0.34 by *t* test; *n* = 23 kaolin; *n* = 16 saline). ***C***, Time taken to escape Barnes maze on multiple trials across 3 d (error bars represent SEM in this figure only; trial effect *p* < 0.0001; treatment effect *p* = 0.033; two-way repeated-measures ANOVA with Geisser–Greenhouse correction; *n* = 20 kaolin; *n* = 15 saline).

To assess recognition memory, we used the novel object recognition test. Saline-injected control mice spent significantly more time with the novel object than chance performance (discrimination index 0.33 ± 0.39 vs 0; *p* = 0.004; Student's *t* test) while hydrocephalic mice did not (discrimination index 0.14 ± 0.40 vs 0; *p* = 0.117; Student's *t* test; *n* = 23 hydrocephalic; *n* = 16 controls; Fig. 6*A*). These results were not confounded by a lack of movement or interest because hydrocephalic mice spent the same amount of overall time interacting with objects as control mice (Fig. 6*B*).

We also assessed memory performance in a visuospatial paradigm. On the Barnes maze, hydrocephalic mice took longer to find the escape port than saline-injected controls (63.68 ± 39.35 vs 33.67 ± 14.87; *p* = 0.033) but both groups improved over time (*p* < 0.0001; *n* = 20 hydro; *n* = 15 controls; Fig. 6*C*). These results indicate that overt ability to encode, store, and recall a spatial location, and ability to walk to that location, all remain intact, but hydrocephalic mice learn the information more slowly.

### Hedonic, affective, and protective behavior

**Figure 7.**
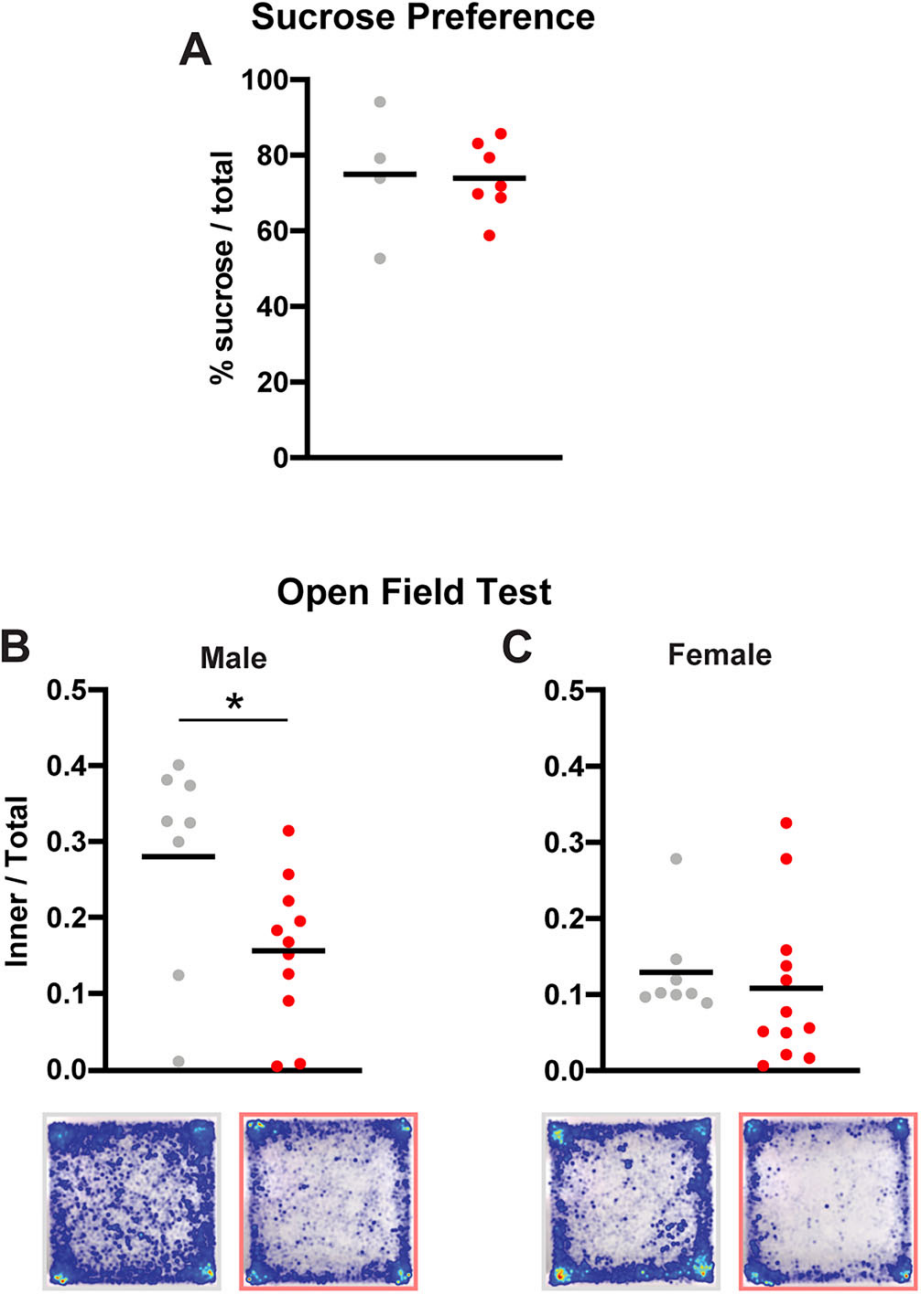
Hedonic and affective behavior. ***A***, Preference for 1% sucrose over water on a 24 h, two-bottle choice test for control (gray, *n* = 4) and hydrocephalic (red, *n* = 7) mice (*p* = 0.89, *t* test). ***B***, Fraction of time spent in the middle 60% of an open field arena for male control (*n* = 8) and hydrocephalic (*n* = 11) mice (*p = 0.034 by *t* test). Below, heatmaps showing the locations of all control (left) and all hydrocephalic (right) male mice. ***C***, Fraction of time spent in middle 60% of an open field arena for female control (*n* = 8) and hydrocephalic mice (*n* = 12, *p* = 0.61 by *t* test). Below, heatmaps showing the locations of all control (left) and all hydrocephalic (right) female mice.

Apathy is an occasional symptom of human NPH (Devito et al., 2005; Kito et al., 2009), and hydrocephalic mice seemed to resist handling less than control mice. While hydrocephalic mice were not overtly apathetic on any behavioral tests above, we ran additional tests to assess affect. First, we used the sucrose preference test (Liu et al., 2018) to assess hydrocephalic mice for anhedonia and apathy. In this test, all mice preferred 1% sucrose to water, and there was no difference between hydrocephalic and control mice (*p* = 0.89; *n* = 7 hydrocephalic; *n* = 4 controls; Fig. 7*A*).

Next, we analyzed open field data for thigmotaxis. Control females spent less time in the center of the apparatus than control males, so we analyzed the sexes separately. While all mice preferred the edges and corners, male hydrocephalic mice spent less time in the center of the arena than male control mice (0.16 ± 0.10 vs 0.28 ± 0.14; *p* = 0.034; *n* = 11 hydrocephalic; *n* = 8 controls; Fig. 7*B*). There was no difference in females (*p* = 0.61; *n* = 12 hydrocephalic; *n* = 8 controls; Fig. 7*C*).

**Figure 8.**
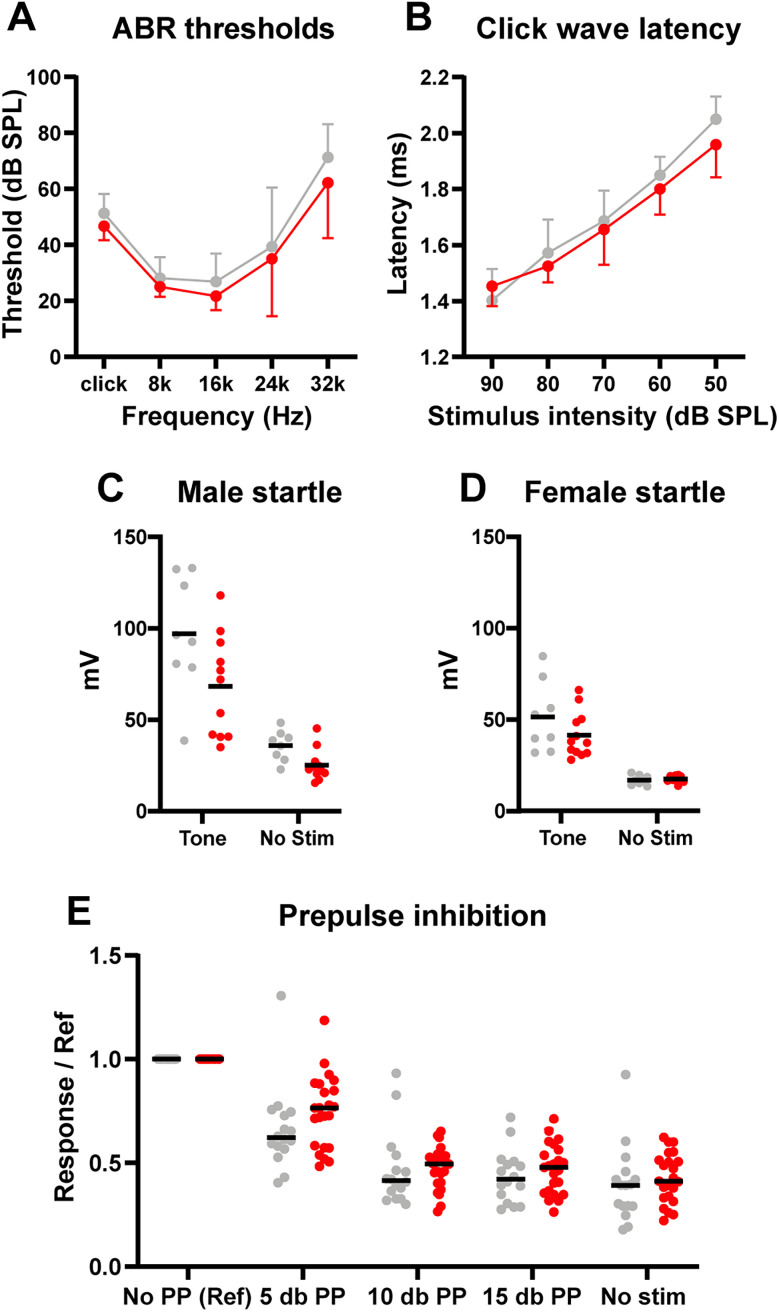
Hydrocephalic mice had normal hearing and startle responses. On auditory brainstem response (ABR) testing, (***A***) hearing thresholds (dB) at different frequencies for control (gray, *n* = 8) and hydrocephalic (red, *n* = 9) mice (*p* = 0.15 click; *p* = 0.31 8k; *p* = 0.21 16k; *p* = 0.67 24k; *p* = 0.27 32k; *t* = test). ***B***, Latency between auditory click stimulus and ABR wave peak across different sound pressure level (SPL) stimulus intensities (*p* = 0.29 90 dB; *p* = 0.32 80 dB; *p* = 0.60 70 dB; *p* = 0.22 60 dB; *p* = 0.12 50 dB; *t* test). ***C***, Male startle response amplitudes following a 120 dB tone or no auditory stimulation (*p* = 0.054 tone; *p* = 0.015 no stim; *n* = 11 kaolin; *n* = 8 saline; *t* test). ***D***, Female startle response amplitudes following a 120 dB tone or no auditory stimulation (*p* = 0.18 tone; *p* = 0.46 no stim; *n* = 12 kaolin; *n* = 8 saline; *t* test). ***E***, Normalized response (divided by reference of response to 120 dB tone for each mouse) to tone following different prepulse intensities for male and female mice (*p* = 0.13 5 dB; *p* = 0.54 10 dB; *p* = 0.25 15 dB; *p* = 0.40 no stim; *n* = 23 kaolin; *n* = 16 saline; *t* test).

We also tested startle response to unpredicted stimuli. Subarachnoid CSF communicates with inner-ear fluid (Salt and Hirose, 2018), and hydrocephalus may cause hearing loss (Sammons et al., 2009), so before testing acoustic startle responses, we evaluated hearing. ABR testing revealed that hydrocephalic mice had the same sound pressure level (SPL) threshold as saline-injected control mice across a variety of frequencies (*n* = 9 hydrocephalic; *n* = 8 controls; Fig. 8*A*). Further, there was no difference in the latency from an auditory click stimulus to the ABR wave peak across multiple intensities (Fig. 8*B*).

Finding no auditory deficit, we next tested acoustic startle response. Control females (*n* = 8) had a lower response amplitude to a loud (120 dB) tone than control males (51.45 ± 19.34 vs 97.01 ± 32.25 mV; *p* = 0.0041; *n* = 8), likely due to size differences, so we analyzed sexes separately. There was no difference in startle response amplitude between hydrocephalic (*n* = 12 female, 11 male) and control mice of either sex, although male hydrocephalic mice had slightly smaller movement amplitudes in the no-stimulation condition (hydro 25.22 ± 8.60 vs saline 35.92 ± 8.29 mV; *p* = 0.015; Fig. 8*C*). Finally, we normalized startle responses to test prepulse inhibition (PPI). Between sexes and between hydrocephalic and control mice, there were no differences in prepulse inhibition of the acoustic startle response (Fig. 8*E*).

Overall, these results indicate a possible impact of hydrocephalus on affect in male but not female mice, but no anhedonia or apathy and no deficit in protective responses to unexpected or preinhibited stimuli.

### Hydrocephalic mice transiently consume less food and lose weight

**Figure 9.**
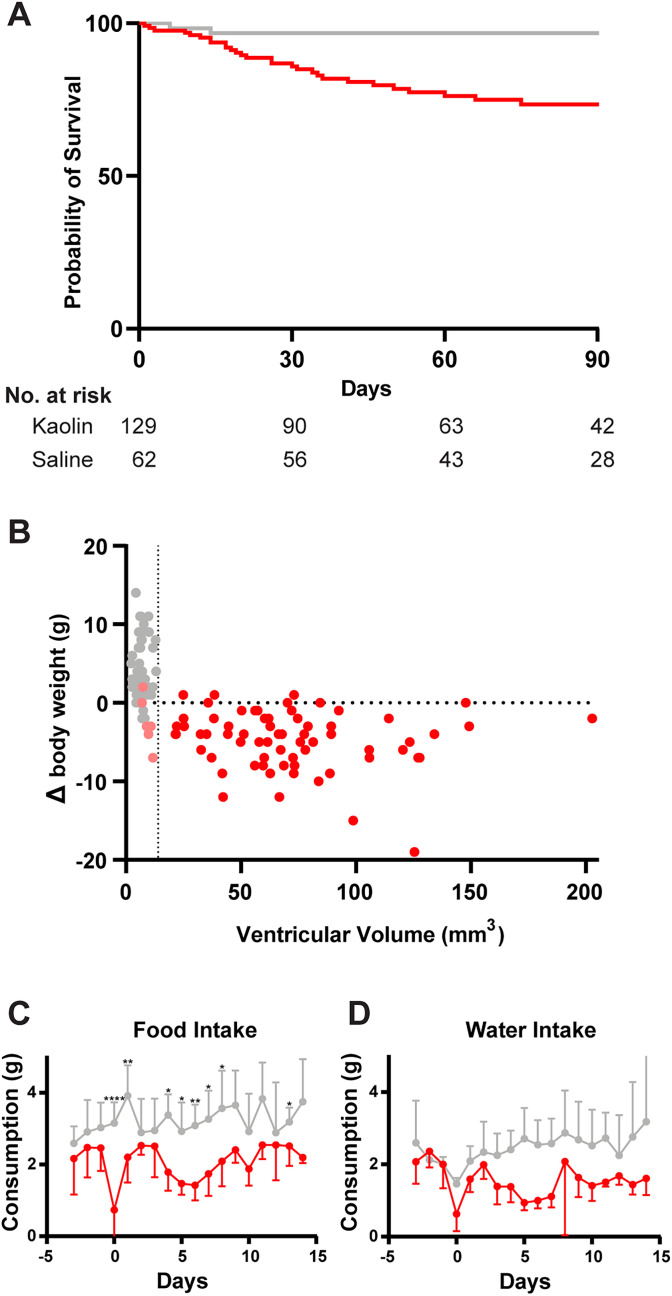
Hydrocephalic mice transiently consume less food and lose weight. ***A***, Kaplan–Meier plot showing survival of saline-injected (gray, *n* = 63) and kaolin-injected (red, *n* = 128) mice. The two saline-injected control mice that were either killed (*n* = 1) or died unexpectedly (*n* = 1) were pair-fed controls in the experiment shown in Extended Data Figure 9-1. Bottom, number (No.) at risk includes all mice remaining in the study that have not reached their experimental endpoint. ***B***, Change in body weight (from day of surgery to day of perfusion) versus cerebroventricular volume on final MRI (*p* = 0.24; *n* = 74 kaolin; *n* = 47 saline). ***C***, BioDAQ food consumption in the first 2 weeks after saline (*n* = 5) versus kaolin (*n* = 6) injection (day factor *p* < 0.0001; treatment factor *p* = 0.0046; treatment × time *p* = 0.017; two-way repeated-measures ANOVA; d0 *p* < 0.0001; d1 *p* = 0.004; d4 *p* = 0.010; d5 *p* = 0.030; d6 *p* = 0.006; d7 *p* = 0.018; d8 *p* = 0.025; d14 *p* = 0.015; Sidak's multiple-comparisons correction) ***D***, BioDAQ water consumption in the first 2 weeks after saline versus kaolin injection (day factor *p* = 0.039; treatment factor *p* = 0.020; treatment × time *p* = 0.005; two-way repeated-measures ANOVA). Extended data on food intake and body weight is shown in Extended Data Figure 9-1.

Injecting larger amounts of kaolin (20–25%) into the cisterna magna has been reported to cause nasal discharge and weight loss in rats (Li et al., 2008) and high mortality in mice during the first week after injection (Bloch et al., 2006; Lopes Lda et al., 2009). None of our kaolin-injected mice had nasal discharge, but most lost weight, and some mice had reduced skin turgor with a rough coat and slightly hunched posture. Occasional male mice developed penile prolapse, which resolved within 1–2 weeks of treatment with antibiotic ointment. Only three of 128 kaolin-injected mice died in the first week. Most mice improved within the first 2 weeks, but 18 (14%) died or had to be killed in the first 30 d. After 30 d, 13 additional mice were killed or died unexpectedly (Fig. 9*A*). Most survived, without complication, for up to 180 d, but unplanned endpoints occurred at seemingly random time points as late as 174 d after injection, when most mice had already met their planned experimental endpoint. Except for two control mice that died during the pair-feeding experiment, no vehicle-injected mice ever appeared ill or died unexpectedly.

10.1523/ENEURO.0412-24.2024.f9-1 Figure 9-1 Pair-fed control mice lost the same amount of weight as kaolin-injected mice. **(A)** Top: food consumed by control (gray, n = 7) and kaolin-injected (red, n = 7) mice on grid-bottom cages from 9 days before surgery to 21 days after surgery, measured every 3 days (time factor p < 0.0001, treatment factor p = 0.006, treatment x time p < 0.0001, mixed-effects model; d0-3 p < 0.0001, d3-6 p = 0.008, d6-9 p = 0.048, d9-12 p = 0.046, Sidak’s multiple comparison correction). Middle: Change in body weight for the same mice (time factor p < 0.0001, treatment factor p = 0.007, treatment x time p < 0.0001, mixed-effects model; d0-3 p < 0.0001, d3-6 p = 0.017, Sidak’s multiple comparison correction). Bottom: Average body weight of the same mice. **(B)** Top: food consumed by pair-fed saline-injected (n = 6) and non-surgical (n = 6) control mice (dark gray, n = 12) vs. kaolin-injected mice (red, n = 6) on grid-bottom cages from 9 days before surgery to 24 (kaolin injected) or 30 (pair-fed) days after surgery, measured every 3 days. Middle: Change in body weight for the same mice. Bottom: Average body weight of the same mice. Download Figure 9-1, TIF file.

Hydrocephalic mice lost weight, but their weight loss did not correlate with cerebral ventricular volume (Fig. 9*B*). Weight loss occurred only in the first week, before hydrocephalus developed (Extended Data Fig. 9-1*A*). By continuously measuring food and water consumption, we observed that kaolin-injected mice (*n* = 6) consumed less food than saline-injected controls in the initial 2 weeks after kaolin injection (2.09 ± 0.50 vs 3.21 ± 0.38 g/d; *n* = 5; day factor *p* < 0.0001; treatment factor *p* = 0.0046; treatment × time *p* = 0.017). Hydrocephalic mice ate significantly less, primarily on the day of surgery but also on the day after surgery and Days 4–8, but they did not consume less food than saline-injected controls on Days 2 and 3 (d0 *p* < 0.0001; d1 *p* = 0.004; d4 *p* = 0.010; d5 *p* = 0.030; d6 *p* = 0.006; d7 *p* = 0.018; d8 *p* = 0.025; Fig. 9*C*). Kaolin-injected mice also consumed less water overall than control mice in the 2 weeks following surgery (1.55 ± 0.45 vs 2.45 ± 0.39 ml/d; day factor *p* = 0.039; treatment factor *p* = 0.020; treatment × time *p* = 0.005; Fig. 9*D*), but this difference was not statistically significant on any individual day.

Weight loss can be caused by reduced caloric intake, increased metabolic activity, or both. To test the mechanism of weight loss after kaolin injection, we ran a pair-feeding (yoked-control) experiment. First, we replicated the previous experiment by measuring *ad libitum* food intake and body weight every 3 d, again finding a transient reduction in food consumption and body weight in kaolin-injected but not saline-injected mice (*n* = 7 each; Extended Data Fig. 9-1*A*). Next, we used the same setup to pair-feed control mice (1:1 saline-injected *n* = 6 and 1:1 unoperated *n* = 6) the same amount of food that had been consumed by kaolin-injected mice (*n* = 6) in the previous 3 d. Pair-fed control mice lost the same amount of body weight as kaolin-injected mice (Extended Data Fig. 9-1*B*). Upon returning to *ad libitum* food availability (Day 24), control mice greatly increased food consumption for the first 3 d and gained back all of the lost weight (Extended Data Fig. 9-1*B*). Overall, these results show that the initial weight loss of kaolin-injected mice can be fully accounted for by their transiently reduced food consumption.

### Histopathology and scanning electron microscopy

**Figure 10.**
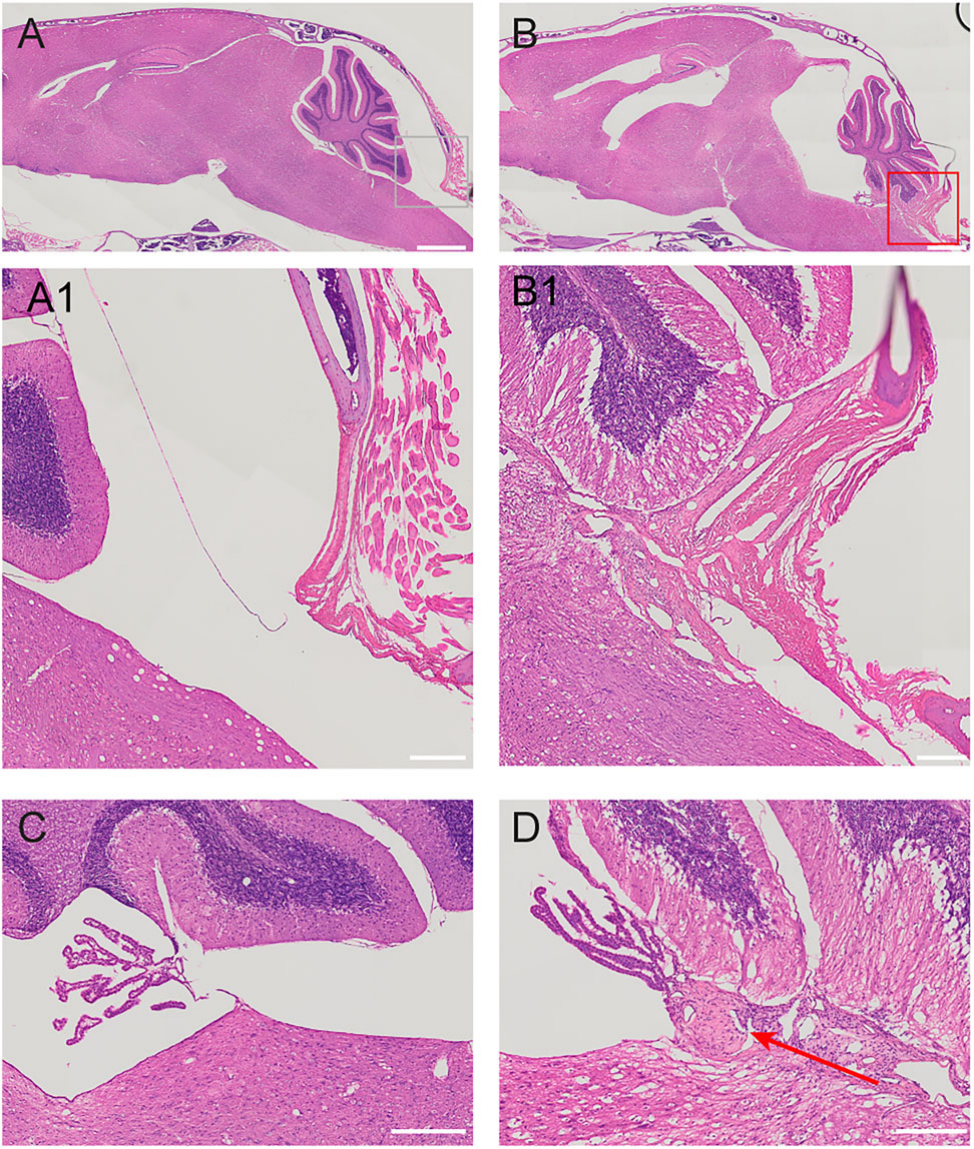
Kaolin-induced hydrocephalus with fibrotic response and granulomatous inflammation. ***A***, Hematoxylin and eosin (H&E) sagittal section from a mouse injected with saline (*n* = 2). Scale bar, 1 mm. ***A1***, Magnified image of the cisterna magna. Scale bar, 200 µm. ***B***, Sagittal section from a mouse injected with kaolin (*n* = 3). Scale bar, 1 mm. ***B1***, Magnified image of the cisterna magna, showing fibrotic response deep to the atlanto-occipital membrane. Scale bar, 200 µm. ***C***, Fourth ventricular choroid plexus in mouse injected with saline. Scale bar, 200 µm. ***D***, Granulomatous inflammation at the root of the choroid plexus, near the obex, in continuity with fibrotic response deep to the atlanto-occipital membrane, in a mouse injected with kaolin. Scale bar = 200 µm.

To investigate the possibility of chronic inflammatory changes in kaolin-injected mice, we performed hematoxylin and eosin (H&E) staining on parasagittal tissue sections from whole mouse heads. We found leptomeningeal fibrosis behind the cerebellum, deep to the site of kaolin injection (*n* = 3 hydrocephalic mice; Fig. 10*B*), similar to previous descriptions in rats (Del Bigio, 2001; Del Bigio et al., 2003; Khan et al., 2006). We did not find this pathology in saline-injected control mice (*n* = 2; Fig. 10*A*). This fibrotic response, along with variable leptomeningeal thickening in the basal cisterns, made gross dissection of the brainstem challenging. In the caudal fourth ventricle, along the root of the choroid plexus, we found granulomatous inflammation (Fig. 10*D*), which was not present in control mice (Fig. 10*C*). Although others have mentioned kaolin deposits or “kaolinomas” that consist of phagocytes with intracellular mineral inclusions (Slobodian et al., 2007; Wang et al., 2011; Hagen et al., 2022), we did not find any in our hydrocephalic mice.

**Figure 11.**
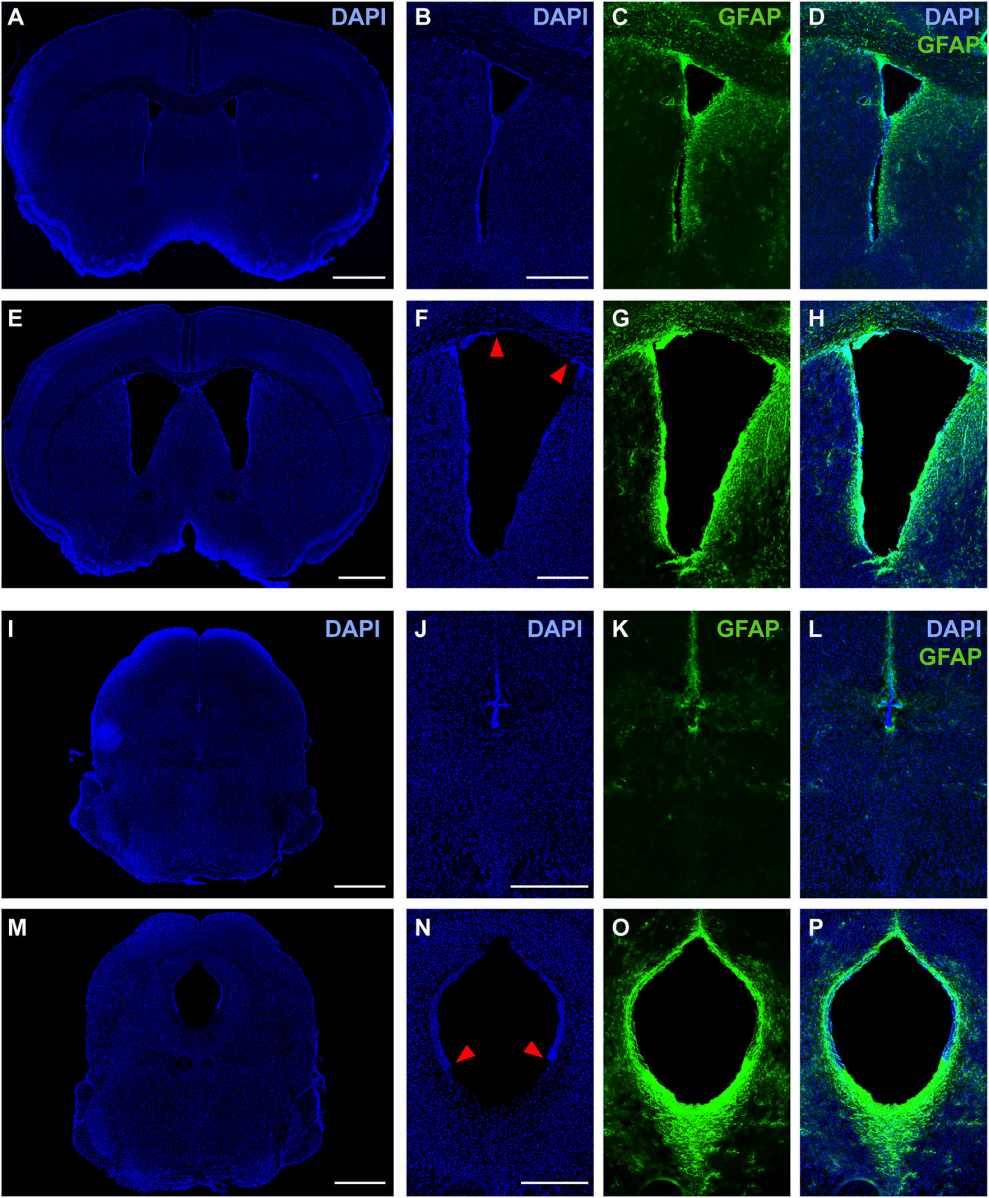
Hydrocephalic mice have increased astrocytic presence near ventricles. ***A***, DAPI-stained axial section from a representative control mouse (saline-injected, *n* = 8) at a level ∼0.5 mm rostral to bregma. ***B–D***, Zoomed-in images of the left lateral ventricle. ***B***, DAPI-stained image showing normal, intact ependymal lining. ***C***, GFAP immunofluorescence (green) shows reactivity in ependyma and light astrocytic presence near ventricle. ***D***, Overlay of DAPI and GFAP. ***E***, DAPI-stained section from a representative hydrocephalic mouse (kaolin-injected, *n* = 11). ***F–H***, Zoomed-in image of the left lateral ventricle. ***F***, DAPI-stained image showing tears in the ependymal lining (red arrowheads). ***G***, GFAP immunolabeling (green) shows reactivity in ependyma and increased astrocytic presence near ventricle, relative to control. ***H***, Overlay of DAPI and GFAP. ***I***, DAPI-stained section of a control mouse at the caudal end of the cerebral aqueduct. ***J–L***, Zoomed-in images of aqueduct. ***J***, DAPI image showing ependyma lining the barely visible aqueduct. ***K***, GFAP immunolabeling shows reactivity in ependyma and very light astrocytic presence near aqueduct. ***L***, Overlay of DAPI and GFAP. ***M***, DAPI-stained section of a hydrocephalic mouse at the caudal end of the cerebral aqueduct. ***N–P***, Zoomed-in images of aqueduct. ***N***, DAPI image showing large cerebral aqueduct with tears in the ventral ependymal lining (red arrowheads). ***O***, GFAP immunolabeling shows reactivity in ependyma with heavy astrocytic infiltration in the midbrain, immediately ventral to the torn ependyma (*p* = 0.0012 by Student's *t* test comparing periaqueductal GFAP intensity in *n* = 11 hydrocephalic vs *n* = 8 saline). ***P***, Overlay of DAPI and GFAP.

We also observed subependymal gliosis and granular ependymitis in hydrocephalic mice. To investigate this further, we performed immunofluorescence labeling for glial fibrillary acidic protein (GFAP), a marker of activated astrocytes. GFAP immunoreactivity highlighted prominent differences in select periventricular regions of hydrocephalic and control mice (Fig. 11). Exclusively in hydrocephalic mice, we found tears in the ependymal lining along the walls of both lateral ventricles (8 of 11 mice examined) and near the caudal end of the cerebral aqueduct, where it expands into the fourth ventricle (10 of 11 mice examined; Fig. 11*N*). In these regions, we found prominent GFAP-immunoreactive processes (*n* = 11 hydrocephalic; *n* = 8 control; Fig. 11*G*,*O*), confirming the subependymal gliosis identified after H&E staining. GFAP labeling in this region was not prominent in saline-injected control mice (Fig. 11*C*,*K*), and the average pixel intensity of GFAP immunofluorescence bordering the caudal cerebral aqueduct was higher in hydrocephalic mice than in controls (24.73 ± 10.61 vs 9.57 ± 3.21; *p* = 0.0012; *n* = 11 hydrocephalic vs *n* = 9 control mice). These regions with GFAP-immunoreactive subependymal gliosis also contained prominent immunoreactivity for aquaporin-4, a water channel in astrocytic end feet (data not shown). Qualitative, whole-brain analysis of additional immunofluorescence markers for neurons, microglia, myelin, and blood vessels did not reveal any prominent or consistent differences in the brain parenchyma of hydrocephalic relative to control mice (data not shown).

**Figure 12.**
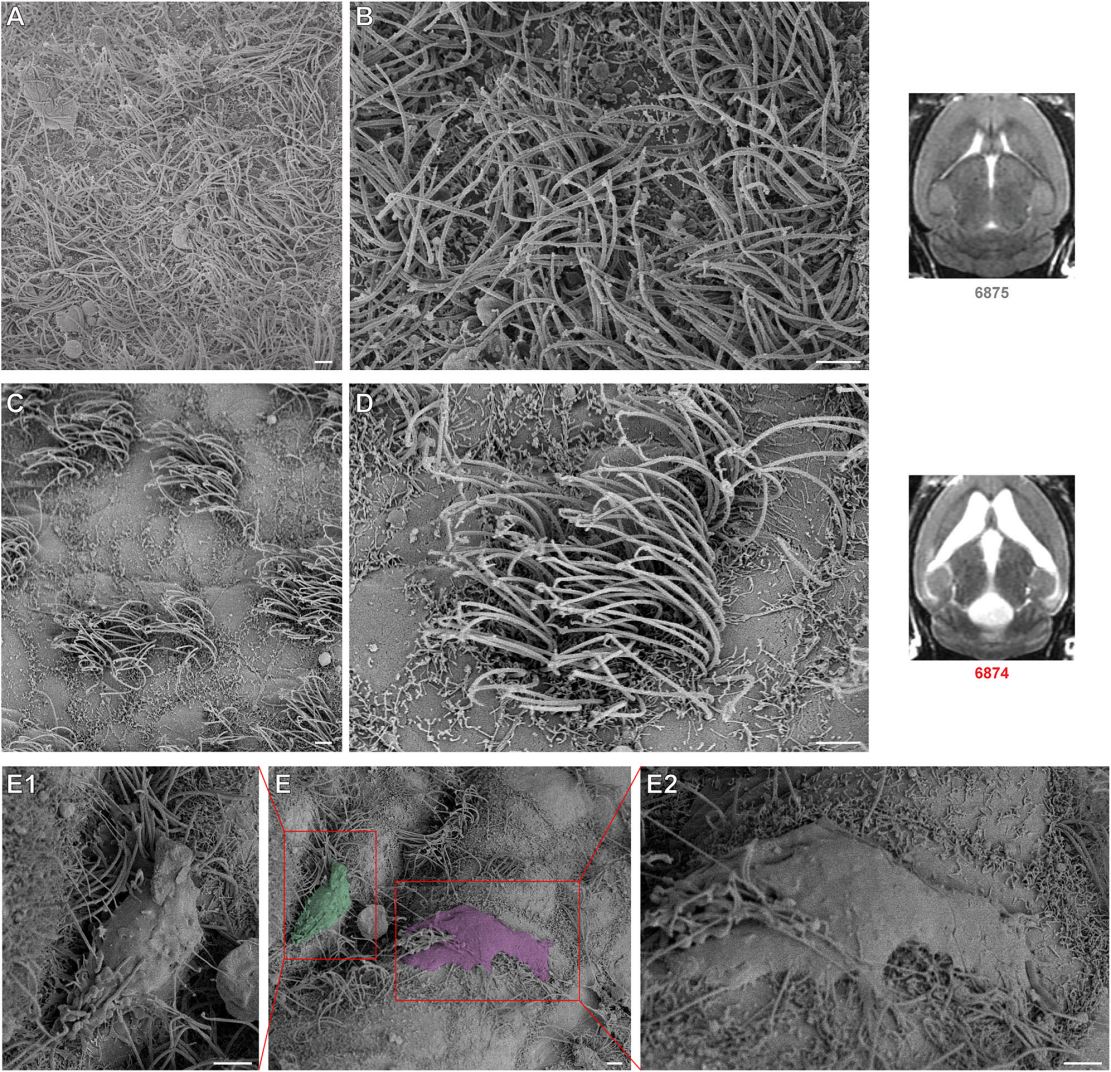
Hydrocephalic mice have more dispersed cilia. ***A***, Floor of the lateral ventricle in saline-injected (control, *n* = 2) mouse. ***B***, Zoomed-in image of ***A*** showing ependymal cell surfaces obscured by thick ciliary cover. ***C***, Floor of the lateral ventricle in kaolin-injected (hydrocephalic, *n* = 3) mouse, showing increased distance between groups of cilia compared with control. ***D***, Zoomed-in portion of ***C***, showing edges of ependymal cell with a single bunch of cilia in the middle of the cell. ***E***, Fourth ventricle of hydrocephalic mouse with abnormal cells highlighted in green and red. ***E1***, ***E2***, Magnified images from of phagocytic cells that appear to be engulfing cilia. All error bars are 2 µm.

Finally, we used scanning electron microscopy (SEM) to examine the fine structure of cellular components along the ventricular walls in hydrocephalic and control mice (*n* = 3; *n* = 2). The most prominent difference was a reduced density of cilia along the lateral ventricles of hydrocephalic mice, as reported in rats and rabbits (Collins, 1979; Torvik et al., 1981). In control mice, ependymal walls were covered in a dense carpet of cilia, but in hydrocephalic mice, tufts of cilia were surrounded by flat surfaces partly covered with microvilli (Fig. 12*A–D*). It appeared that individual ependymal cells were stretched, with a tuft of cilia remaining in the center, while control mice had smaller cells, fully covered by cilia. Along the floor of the fourth ventricle in hydrocephalic mice, we also found rare cells that appeared to be phagocytes (Fig. 12*E*).

**Movie 1.** Rotating 3D rendering of cerebral ventricles from control (gray) and hydrocephalic (red) mice. [View online]

**Movie 2.** Representative examples of control (left) and hydrocephalic (right) gait on a clear treadmill moving at 10 cm/s. [View online]

**Movie 3.** Voiding behavior in a representative control mouse and an incontinent hydrocephalic mouse. Control mouse stops moving to void in upper-left edge and returns to sniff void. Void cools over the first minute and is stable at 5 and 30 min. Hydrocephalic mouse voids multiple times in a line while walking, following a void in the center of the enclosure. By 5 min, it is difficult to see the small line of voids, which are thermally invisible by 30 min. At 30 min, the mouse has another incontinent void while walking. [View online]

## Discussion

An ideal animal model for NPH would have chronic, communicating hydrocephalus and normal intracranial pressure. It would also have gait dysfunction, cognitive impairment, and urinary incontinence. Our mouse model complements previous animal models that share some of these features and is the first NPH model with urinary frequency and incontinence. The most important advantage of this model is that it can be combined readily with the wide variety of genetic strains and molecular tools available for preclinical experiments in mice. NPH co-occurs with a variety of other diseases, and implementing this NPH model in mouse models of comorbid human diseases will enable progress in understanding disease interactions. Importantly, this mouse model will be useful for identifying the neural circuit mechanisms of each NPH symptom, which will enable development of new therapies for shunt-resistant symptoms.

### Preclinical models

Over a century ago, Dandy and Blackfan produced noncommunicating (obstructive) hydrocephalus by blocking the cerebral aqueduct in dogs (Dandy and Blackfan, 1913). Since that time, investigators have used a variety of additional methods to produce noncommunicating hydrocephalus in several species. Some injected blood or bacteria into the lateral ventricle (Aquilina et al., 2008; Yung et al., 2011; Ahn et al., 2013, 2020; Gao et al., 2014; Pan et al., 2023). Others obstructed the cerebral aqueduct or used toxins to cause congenital aqueductal stenosis (De, 1950; Stempak, 1964; Agnew et al., 1968; Milhorat et al., 1970; Jones and Bucknall, 1988; Johnson et al., 1999). None of these animal models resemble NPH, which is a form of communicating hydrocephalus.

Communicating (nonobstructive) hydrocephalus has been induced using other methods, but each has important limitations. Various congenital models develop hydrocephalus with a wide range of severity (Borit and Sidman, 1972; Jones, 1985; Bruni et al., 1988; Dahme et al., 1997; Davy and Robinson, 2003; Crews et al., 2004). Most do not survive to adulthood, and congenital models are not ideal for studying NPH because it is an adult-onset disease. Furthermore, the typically recessive mutations in these congenital models are cumbersome to breed in combination with mouse models that would be useful for exploring effects of hydrocephalus comorbidity in other diseases (Blanchard et al., 2003; Dauer and Przedborski, 2003; Oakley et al., 2006) or with recombinase-conditional mouse strains that are useful for studying specific cell types (Daigle et al., 2018). In contrast, our approach can be implemented in any mouse strain.

A noncongenital model is ideal, but incomplete understanding of what exactly causes chronic, communicating hydrocephalus in human patients has limited progress in this direction. A consistent finding in the preclinical literature is that silicon-containing substances—primarily kaolin (aluminum silicate) or silicone oil (polydimethylsiloxane)—can cause hydrocephalus (Wisniewski et al., 1969; L. K. Page and White, 1982; Del Bigio and Bruni, 1987). One study in rats found that other compounds either did not produce hydrocephalus or caused high mortality; only kaolin consistently produced hydrocephalus (Slobodian et al., 2007). Similarly, in our study, injecting blood or LPS was unsuccessful, whereas kaolin produced hydrocephalus in over 90% of mice.

Kaolin has been used to produce hydrocephalus in a variety of animal species (Weller and Wiśniewski, 1969; Hochwald et al., 1973, 1975, 1981; Del Bigio et al., 1997; Bloch et al., 2006; Lopes Lda et al., 2009; McAllister et al., 2021). Injecting kaolin into the cisterna magna or other cisternal spaces reliably caused hydrocephalus, while injecting it into subarachnoid spaces overlying the cerebral hemispheres in rats had a less pronounced effect (Cosan et al., 2002; Li et al., 2008; Jusué-Torres et al., 2016). Prior to our study, kaolin had been used in a handful of mouse studies, many of which focused on neonates (Femi-Akinlosotu et al., 2019; Femi-Akinlosotu and Shokunbi, 2020; F. Olopade et al., 2022). Other studies reported high mortality the week after injection in adult mice (Bloch et al., 2006; Lopes Lda et al., 2009). We improved survival by delivering less kaolin (5 µl of a 15% suspension, vs typically 10 µl of 25% in previous studies), and >90% of our mice survived the first week, when inflammatory responses to kaolin seem maximal. One study reported using less kaolin (5 µl of 2%) but did not find a behavioral change (Hatta et al., 2006). Our pilot injections with less kaolin did not produce hydrocephalus consistently, and we found that 5 µl of 15% kaolin produced an optimal balance between efficacy and survival.

### Kaolin causes transient inflammation followed by communicating hydrocephalus

Our approach relies on a stimulus that initially causes inflammation (Jeunesse et al., 2011; Camargo et al., 2015; Levionnois et al., 2018; Vargas-Ruiz et al., 2020; Wiemann et al., 2020), raising the possibility that early effects are secondary to an inflammatory response, rather than hydrocephalus. Indeed, our analysis of food intake identified a transient decrease in appetite followed by normalization of food intake before hydrocephalus becomes apparent. Months later, the primary changes evident in the brain parenchyma were disruption of the ependymal lining of the lateral ventricle and cerebral aqueduct, with focal, periventricular gliosis. Similar findings were reported in noninflammatory animal models (Milhorat et al., 1970; Miller and McAllister, 2007) and in human NPH patients (Eide and Hansson, 2018). Along the ependymal surface, we found rare macrophages that appeared to be engulfing cilia, as reported previously in rats and rabbits (R. B. Page, 1975; Go et al., 1976). Within the brain parenchyma, however, we did not observe microgliosis or neuronal loss.

The initial inflammatory response to kaolin may lead to a CSF-resorptive deficit that causes hydrocephalus. While it is often idiopathic, roughly half of NPH patients develop hydrocephalus after meningitis, subarachnoid hemorrhage, neurosarcoidosis, or other inflammatory etiologies (Damasceno, 2015; Daou et al., 2016; Owens et al., 2021). Much like sarcoidosis, kaolin causes granulomatous inflammation, which consists of clustered macrophages with variable associated lymphocytic infiltrates. This type of inflammatory response could be responsible for CSF-resorptive deficits in a subset of communicating hydrocephalus cases.

Some investigators have claimed that kaolin causes noncommunicating (obstructive) hydrocephalus by blocking CSF outflow from the fourth ventricle (Hochwald et al., 1975; McAllister and Chovan, 1998; F. E. Olopade et al., 2012). However, the foramina of Luschka remained patent in our mice, and others have reported similar findings (Bloch et al., 2006; Li et al., 2008). Further, our observation of an enlarged subarachnoid space dorsal to the cerebral hemispheres in some mice is similar to the disproportionately enlarged subarachnoid space in hydrocephalus (DESH) in some NPH patients (Hashimoto et al., 2010). This observation further suggests that decreased CSF absorption, rather than ventricular obstruction, caused hydrocephalus in kaolin-injected mice. Testing this hypothesis will require additional experiments that assess CSF outflow in this model.

### Intracranial pressure

Normal intracranial pressure is a sine qua non for a model of NPH. In our hydrocephalic mice, the average intracranial pressure was normal. A transient rise in ICP has been reported in the hours to days following kaolin injection (Cosan et al., 2002; Kondziella et al., 2002; Bloch et al., 2006; Shulyakov et al., 2012; Hagen et al., 2022), but we did not find a difference in ICP between hydrocephalic mice and control mice across >2 months. Future methods for continuous ICP monitoring in freely behaving mice may reveal additional information about CSF pressure dynamics in this disease model.

### NPH symptoms in mice and men

The first and most common stereotypical symptom of NPH is gait impairment. Gait deficits have been reported in a variety of juvenile and adult hydrocephalic animal models (Del Bigio et al., 1997; Lopes Lda et al., 2009; F. E. Olopade et al., 2012; Zhu et al., 2022). However, these deficits were not always easy to measure, with one study mentioning a qualitatively impaired gait but finding no difference on rotarod (Khan et al., 2006). Here, along with a slight difference in formal DigiGait analysis, we found a clear balance deficit on the rotarod test. Unsteady gait is typically the first symptom in human NPH (Williams and Malm, 2016), and imbalance appeared early in hydrocephalic mice. Interestingly, our hydrocephalic mice did not move less or more slowly in an open field arena, but they did have reduced maximum gait speeds on a treadmill test. This again recalls the human disease, where reduced gait velocity is common and distinguishes NPH from other forms of hydrocephalus (Williams et al., 2019). The specific neural circuit mechanisms of these symptoms remain uncertain, and our novel observation of shaggy cortical tissue in the caudal midline of the cerebellum may be pertinent because this posterior region of the cerebellar vermis is important for vestibular-motor function, which allows smoothly scaled and balanced movements. Exploring this possibility in this mouse model may lead to new questions and treatment opportunities for human gait abnormalities.

The second stereotypical symptom of NPH is cognitive dysfunction. The pattern of cognitive domains affected is variable but frequently includes memory and attention, with variable psychomotor slowing (Ogino et al., 2006; Picascia et al., 2015). Complementing reports that hydrocephalic rats have prolonged reaction times on operant tasks (Kuchiwaki et al., 1994) and prolonged escape times in the Morris water maze (Del Bigio et al., 2003; Khan et al., 2006), hydrocephalic mice in our study exhibited impaired learning. On a novel object recognition test, many did not outperform chance. They also took longer to find the escape port on a spatial memory task, but their performance improved over time, indicating that cortical memory function remained grossly intact. Initial cognitive impairments in human NPH are more subtle than the anterograde amnesia of Alzheimer's disease (Juhlin et al., 2023), and deficits like impaired attention and processing speed are often described as “subcortical.” NPH patients are sometimes described as apathetic (Vanneste, 2000; Devito et al., 2005), and we found an unexpected deficit in male hydrocephalic mice on the open field test, which could be interpreted as anxiety (avoidance of the open space) or apathy (reduced exploration of the center). It is tempting to speculate that the astrogliosis we found beneath the cerebral aqueduct—in the dorsal raphe nucleus, whose serotonergic neurons are implicated in anxiety and arousal responses (Kaur et al., 2020; Venner et al., 2020)—may play a role. The sex difference observed aligns with previous publications that male rodents have a higher anxiety-like phenotype (Domonkos et al., 2017; Scholl et al., 2019; Börchers et al., 2022) and should be investigated in subsequent tests of cognitive impairment in this model. Overall, the fact that we saw only slight impairments on specific tasks, and no impairment on others, recalls the subtle cognitive impairments in human NPH.

The third stereotypical symptom of NPH is urinary frequency leading to urge incontinence. Before the present study, this symptom had not been reported in any animal model of hydrocephalus. To test urinary behavior in mice, we used a noninvasive technique, micturition video thermography (Verstegen et al., 2020). In contrast to gait and balance impairments, which began in the first week, urinary frequency did not appear until 5 weeks after kaolin injection. Also, while most mice exhibited urinary frequency at least once, incontinent voiding behavior was less common. This aligns with human NPH, where urodynamic testing identifies bladder overactivity in most patients, yet overt incontinence is less common and often appears after gait impairment (Sakakibara et al., 2008). Urodynamic experiments in hydrocephalic mice may reveal earlier bladder dysfunction, before the appearance of urinary frequency and incontinent voiding, and our mouse model opens opportunities to investigate neural circuit mechanisms for each of these findings. Previous work on micturition circuitry focused attention on the hypothalamus, frontal cortex, periaqueductal gray, and neurons in the “pontine micturition center” (Barrington's nucleus) that control the bladder and urinary sphincter (Sakakibara et al., 2012; Tish and Geerling, 2020). Key neurons in this circuit lie near the disrupted ependyma and parenchymal astrogliosis we found in hydrocephalic mice, and they or their continence-controlling input connections may be disrupted in NPH. Identifying an NPH disease model with urinary frequency and incontinence opens the door to studying these circuit mechanisms and developing new treatments for this disabling neurologic symptom.

### Is symptom severity related to ventriculomegaly?

An important consideration in the pathophysiology of NPH symptoms is whether they relate to degree of ventriculomegaly. In humans, it has been reported that behavioral impairments and cognitive deficits are not related to degree of ventriculomegaly (Jackson and Lorber, 1984). Kaolin produces a varying degree and pattern of ventriculomegaly, and our large numbers of mice with varying deficits allowed us to directly compare ventriculomegaly to several behavioral deficits. Surprisingly, we did not find a correlation between ventricular size and any behavioral deficit. One previous study in rats reported different decreases in grip strength and ambulation after categorizing kaolin-induced ventriculomegaly as mild, moderate, or severe, without regression analysis (F. E. Olopade et al., 2012). Another study found that while locomotor activity did not correlate to ventricular volume, there was a small, significant correlation between ventricular volume and Morris water maze performance (Del Bigio et al., 2003).

In contrast to acute hydrocephalus, shunt therapy does not decrease ventriculomegaly in most patients with NPH. Clinical improvements are just as good or better in patients with unchanged ventricular volumes (Meier et al., 2003; Meier and Mutze, 2004), with other factors influencing improvement (Mocco et al., 2006). Like these results in humans, our results indicate that the stereotypical symptoms of NPH are not caused by ventricular expansion alone. It will be important to identify the underlying mechanisms because this information is necessary for treating shunt-resistant symptoms. Combining our NPH mouse model with the large array of genetic and molecular tools already available for targeting and testing specific cell types and synaptic connections will accelerate work in this direction.

In conclusion, our mouse model with communicating, NPH develops the three characteristic symptoms of human NPH: gait impairment, cognitive dysfunction, and urinary frequency with incontinence. These symptoms are not explained simply by the degree of ventriculomegaly, and additional work is needed to identify the neural circuit mechanism of each symptom. Implementing our NPH model in the wide variety of mouse strains and molecular-genetic tools available for studying comorbid disease interactions and testing cell type-specific hypotheses will enable preclinical discoveries that facilitate development of new treatments for NPH symptoms.

## Data Availability

The data that support the findings of this study are available from the corresponding author upon reasonable request.

## Synthesis

Reviewing Editor: David Schoppik, New York University Grossman School of Medicine Decisions are customarily a result of the Reviewing Editor and the peer reviewers coming together and discussing their recommendations until a consensus is reached. When revisions are invited, a fact-based synthesis statement explaining their decision and outlining what is needed to prepare a revision will be listed below. The following reviewer(s) agreed to reveal their identity: NONE. Note: If this manuscript was transferred from JNeurosci and a decision was made to accept the manuscript without peer review, a brief statement to this effect will instead be what is listed below.

OK so normally I wouldn't write much here except that in your fairly strident rebuttal you used the phrase "helpful for the career development of junior scientists, including the graduate student first-author of this paper..." I'm going to take a minute and share some thoughts in the hope that they will be useful to said graduate student first-author.

Critique is at the heart of the scientific enterprise. Learning to parse and respond to it is key to your future success. A big part of that is understanding the motivation of your critics. Take me. I'm writing this to you in the evening on a Sunday instead of watching Brooklyn Nine-Nine with my kids because I think it's important. I volunteer my time as an editor for eNeuro because I value the journal and want it to succeed. I've got half a dozen higher priority things I could be working on, but I'm taking time now for you and your manuscript. It would be foolhardy for you to telegraph that you don't respect my time.

When I go looking for reviewers, I spend a fair bit of time finding people that are expert in the field. When they say "yes" they do so because they think that their comments will help strengthen the manuscript. They care enough to take the time to do some unpaid reading and critiquing -- and it's a good few hours of work. Most of them are just as busy as me, if not busier. When they get done making suggestions, *they are invested in the outcome* because of the work they just did. They want to make your paper better.

If they ask for an analysis, or say something to the effect of "this is what you should worry about" the subtext is "a reasonable reader like me would worry about this, and so you should change your manuscript to address that concern." If they ask for the "n" to be clearer, you can make a table. If they ask for multivariate statistical analyses, run the analyses and report the results in the paper. You are welcome to disagree. It's your paper, after all. But if you don't change the manuscript -- even if the new analyses don't show anything interesting and you put them in your rebuttal letter -- it's a big FU to the reviewer. Why? Because you're saying "I don't think anyone else will ever share that concern." I don't see how you could be that confident, but even if you're right, you'll lose their support.

That's how we got here. You chose to respond to the initial round of criticism a certain way; the language the reviewers chose to use is a red herring. This is just my opinion. I hope a bit of perspective will help guide your future choices and that outcomes will improve as a result.
