# Supplementary figures and images for: Normal Pressure Hydrocephalus in Adult Mice Causes Gait Impairment, Cognitive Deficits, and Urinary Frequency with Incontinence
Source: eNeuro. 2024 Nov 26;11(11):ENEURO.0412-24.2024. doi: 10.1523/ENEURO.0412-24.2024 (PMC11595603; doi:10.1523/ENEURO.0412-24.2024)

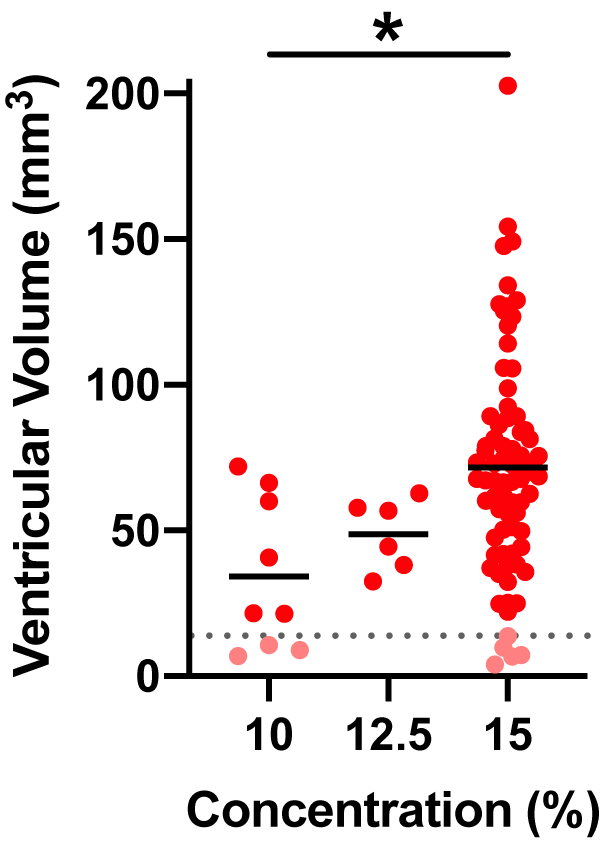

Supplement: Figure 2-1 — Total ventricular volumes measured 6-10 weeks after injecting 15% (n = 75) vs. 12.5% (n = 6) vs. 10% (n = 9) kaolin into the cisterna magna (p = 0.00835 One-way ANOVA, 15% vs 10% p = 0.012 with Tukey’s multiple comparisons correction). Download Figure 2-1, TIF file. [file eneuro-11-ENEURO.0412-24.2024-s004.tif]

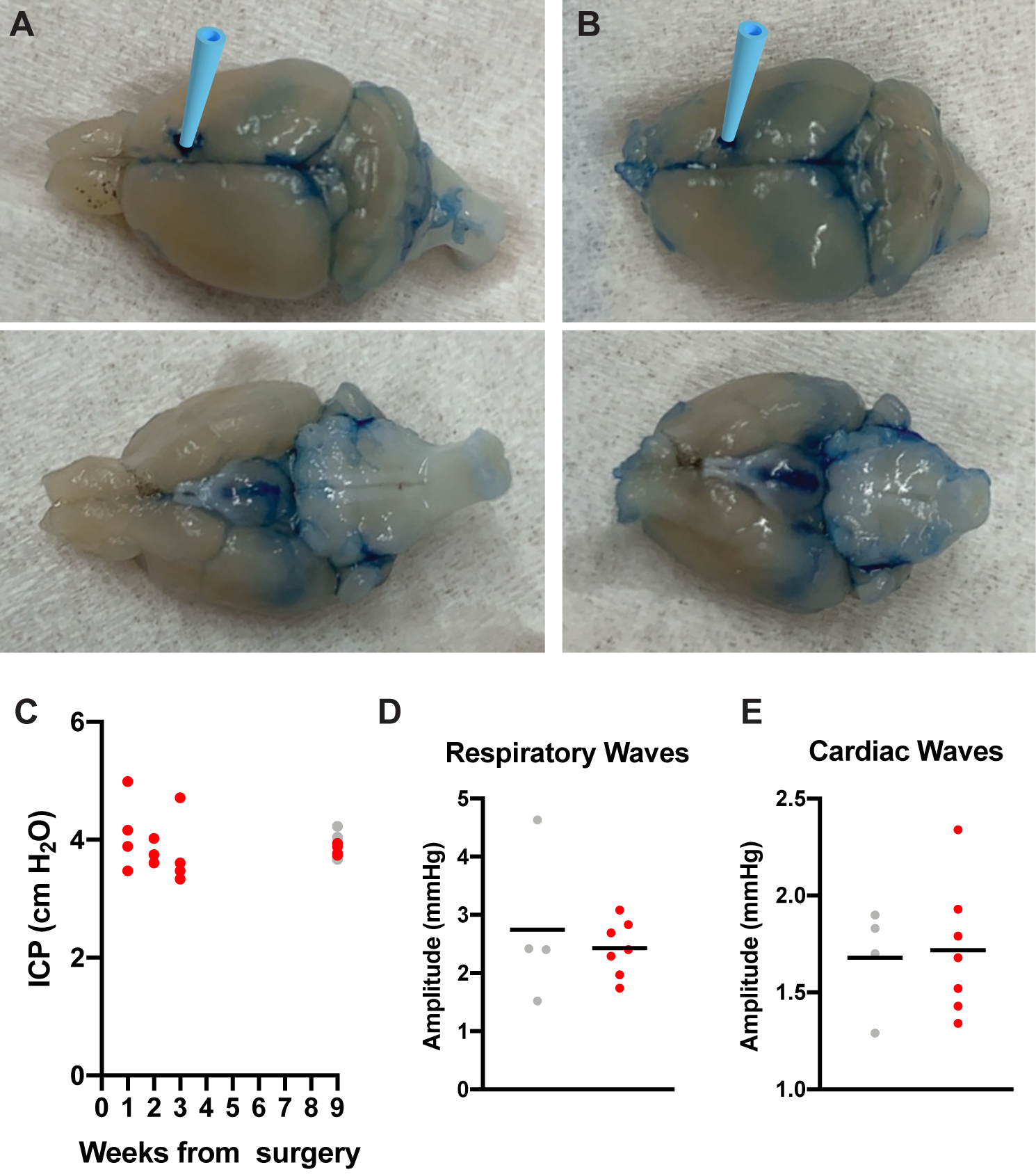

Supplement: Figure 3-1 — (A, B) Dorsal (top) and ventral (bottom) images of two representative brains in which Evans Blue dye was injected into the lateral ventricle 4 weeks after kaolin injection. (C) ICP measurements at different timepoints following kaolin injection (p = 0.68, one-way ANOVA of hydrocephalic groups (n = 4 each) across different time points; p = 0.37 t-test of saline vs. kaolin at week 9, n = 3 saline, n = 4 kaolin). (D–E) Average ICP waveform amplitudes (n = 4 saline, 7 kaolin) of (D) Respiratory (slow) waves (p = 0.57, t-test) and (E) Cardiac (fast) waves (p = 0.85, t-test). Download Figure 3-1, TIF file. [file eneuro-11-ENEURO.0412-24.2024-s005.tif]

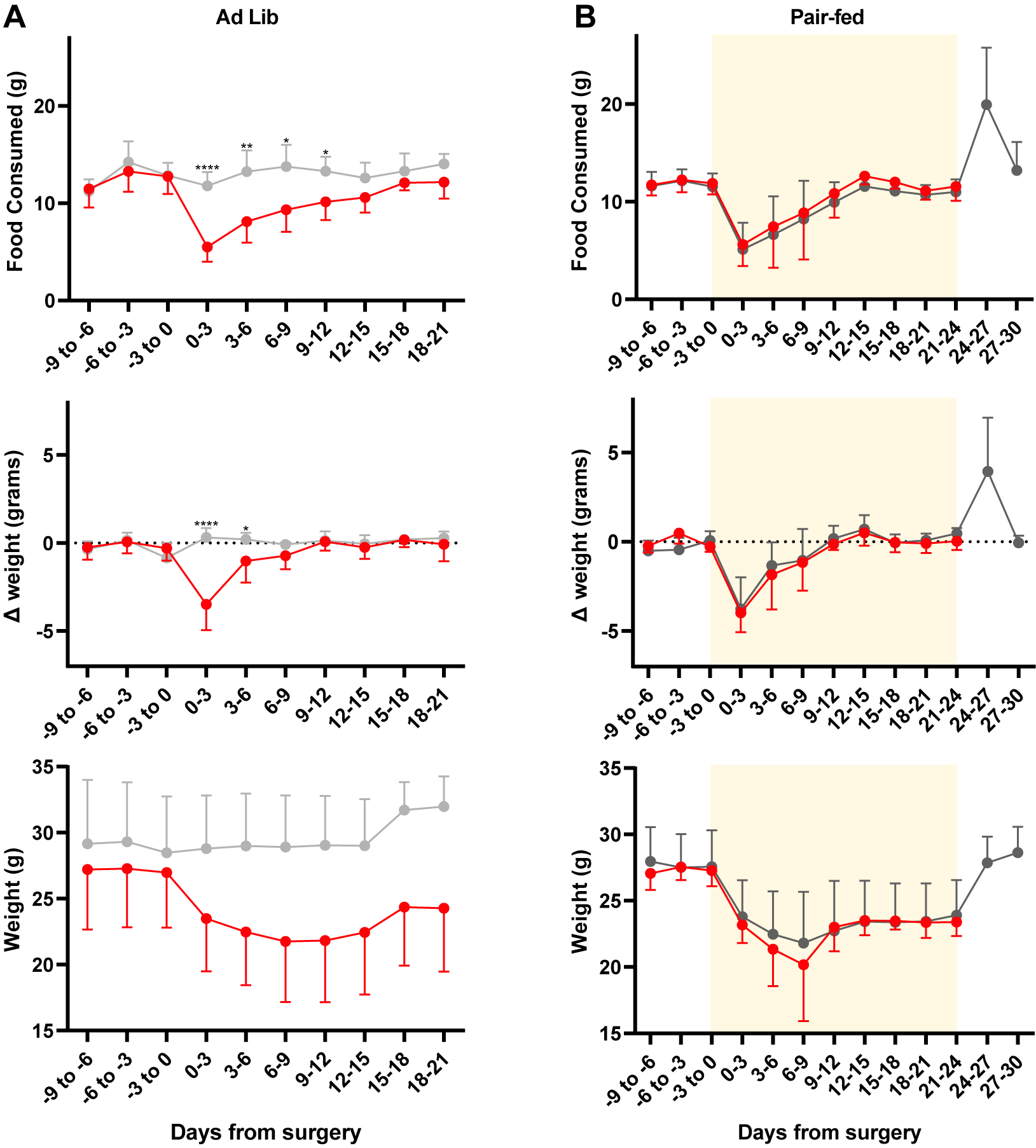

Supplement: Figure 9-1 — Pair-fed control mice lost the same amount of weight as kaolin-injected mice. (A) Top: food consumed by control (gray, n = 7) and kaolin-injected (red, n = 7) mice on grid-bottom cages from 9 days before surgery to 21 days after surgery, measured every 3 days (time factor p < 0.0001, treatment factor p = 0.006, treatment x time p < 0.0001, mixed-effects model; d0-3 p < 0.0001, d3-6 p = 0.008, d6-9 p = 0.048, d9-12 p = 0.046, Sidak’s multiple comparison correction). Middle: Change in body weight for the same mice (time factor p < 0.0001, treatment factor p = 0.007, treatment x time p < 0.0001, mixed-effects model; d0-3 p < 0.0001, d3-6 p = 0.017, Sidak’s multiple comparison correction). Bottom: Average body weight of the same mice. (B) Top: food consumed by pair-fed saline-injected (n = 6) and non-surgical (n = 6) control mice (dark gray, n = 12) vs. kaolin-injected mice (red, n = 6) on grid-bottom cages from 9 days before surgery to 24 (kaolin injected) or 30 (pair-fed) days after surgery, measured every 3 days. Middle: Change in body weight for the same mice. Bottom: Average body weight of the same mice. Download Figure 9-1, TIF file. [file eneuro-11-ENEURO.0412-24.2024-s006.tif]
